# Improved GWO and its application in parameter optimization of Elman neural network

**DOI:** 10.1371/journal.pone.0288071

**Published:** 2023-07-07

**Authors:** Wei Liu, Jiayang Sun, Guangwei Liu, Saiou Fu, Mengyuan Liu, Yixin Zhu, Qi Gao

**Affiliations:** 1 College of Science, Liaoning Technical University, Fuxin, China; 2 Institute of Mathematics and Systems Science, Liaoning Technical University, Fuxin, China; 3 Institute of Intelligent Engineering and Mathematics, Liaoning Technical University, Fuxin, China; 4 College of Mines, Liaoning Technical University, Fuxin, China; 5 College of Civil Engineering, Liaoning Technical University, Fuxin, China; Firat Universitesi, TURKEY

## Abstract

Traditional neural networks used gradient descent methods to train the network structure, which cannot handle complex optimization problems. We proposed an improved grey wolf optimizer (SGWO) to explore a better network structure. GWO was improved by using circle population initialization, information interaction mechanism and adaptive position update to enhance the search performance of the algorithm. SGWO was applied to optimize Elman network structure, and a new prediction method (SGWO-Elman) was proposed. The convergence of SGWO was analyzed by mathematical theory, and the optimization ability of SGWO and the prediction performance of SGWO-Elman were examined using comparative experiments. The results show: (1) the global convergence probability of SGWO was 1, and its process was a finite homogeneous Markov chain with an absorption state; (2) SGWO not only has better optimization performance when solving complex functions of different dimensions, but also when applied to Elman for parameter optimization, SGWO can significantly optimize the network structure and SGWO-Elman has accurate prediction performance.

## 1. Introduction

Elman neural network is a typical local regression network [1]. It has been widely used in the fields of image recognition, fault detection, and big data prediction because of its strong memory capacity and high computational efficiency [2]. The performance of Elman is largely influenced by its training process. Therefore, exploring a high-quality training process has become a key problem to solve in neural network research [3].

In the early 1990s, gradient descent and stochastic methods were the two main Elman training methods [4]. However, gradient descent methods have three main drawbacks [5]: difficulty in finding the global optimal solution, slow convergence, and high dependence on the initial parameters. Similarly, stochastic methods can also weaken the training ability by initializing the parameters. As a result, in the late 1990s, some studies constructed a neural network as a nonlinear optimization model to replace the original linear model [6]. Although this approach avoids computing gradient information, it is not applicable when the dimension exceeds the memory range. Accordingly, starting in 2000, some researchers considered the training network structure as an optimization problem of finding the optimal parameters in a finite space [7]. Some scholars solved this optimization problem by heuristic methods [8]. However, the method needed to increase search space when traversing the set of parameters, which improved the time complexity of the algorithm [9].

To explore better network structures and improve the performance of neural networks, metaheuristic algorithms have become reliable alternatives [10]. Compared to gradient descent methods, metaheuristic algorithms show higher efficiency in avoiding local extremum. These algorithms shift from local search to global search, making them more suitable for global optimization. Therefore, researchers have used metaheuristic algorithms in Elman as an optimization strategy for network structures, and a series of more meaningful results have been achieved so far. For example, Zhang et al. used an improved arithmetic optimizer (IAO) to train the Elman network structure [11]; For the soil salinity prediction problem, the sine cosine algorithm (SCA) was applied to adjusting the parameters of Elman [12], and the experimental results demonstrated that SCA could improve the prediction efficiency of Elman; Some researchers used the particle swarm optimization (PSO) algorithm to optimize Elman parameters and PSO-Elman based on load prediction model [13], compaction density evaluation model [14] and parameter evaluation model were constructed [15]; Metaheuristic algorithms were combined for adjusting the weights and thresholds of Elman. For example, the ant colony algorithm (ACO) and genetic algorithm (GA) were combined to form AGA-Elman [16]; SUN et al. developed an Elman prediction model based on a whale optimization algorithm (WOA) [17]. The experimental results proved that WOA-Elman has good engineering utility the porosity prediction. In addition, WOA-Elman also played an important role in weather prediction [18] and landslide probability prediction [19].

Although various metaheuristic algorithms have been deployed and studied to train Elman, local extremum still exists. The grey wolf optimizer [20] (GWO) is a recently proposed metaheuristic algorithm. GWO is inspired by the wolves hierarchy and the hunting process. GWO has three leaders who are responsible for guiding the wolves to attack, delivering attack information and leading the pack to encircle [21]. During the iterative, the three wolves continuously update their positions and thus search for the global optimum. Due to its few parameters, easy implementation and strong convergence, GWO has shown excellent performance in solving high-dimensional optimization problems [22]. However, the global search capability of GWO is still poor, and it is easy to fall into local extremes. However, the well-known No Free Lunch Theorem [23] states that there is no universal metaheuristic algorithm that can solve all optimization problems. Therefore, our research aims to focus on two points. First, to propose a more efficient improved grey wolf optimizer based on the algorithm characteristics. Second, to explore a better method for training network structures based on the improved grey wolf optimizer.

Therefore, we propose an Elman training method based on the improved grey wolf optimizer (SGWO). SGWO introduces three strategies into the wolf hunting process: circle chaotic mapping, information interaction mechanism and the adaptive position update strategy. We use circle chaotic mapping to increase the population diversity; In the information interaction machine, the head wolf position is perturbed by the Cauchy variation to jump out of the local optimum, and the information transfer between wolves is enhanced by the golden sine algorithm, thus accelerating the convergence of SGWO; Meanwhile, the adaptive position update strategy is used to adjust the search range autonomously, enabling SGWO to balance the global and local searches. In addition, we innovatively introduce the Markov process and probabilistic analysis to demonstrate the convergence performance of SGWO. Ablation experiments based on three strategies are also conducted and SGWO is compared with seven optimization algorithms to analyze the optimization performance of the improved grey wolf optimizer. Based on this, we incorporate SGWO into the Elman training process and construct an SGWO-Elman prediction model. The SGWO-Elman is also compared with three types of algorithms, including Elman neural network based on other optimization algorithms, other neural networks and other neural networks based on SGWO to verify the prediction ability of SGWO-Elman model for complex problems.

The rest of the paper is organized as follows. Metaheuristic algorithms classification and variants of GWO are mentioned in Section 2. Section 3 gives a brief description of the grey wolf optimizer. The improved grey wolf optimizer (SGWO) is introduced and proved in Section 4. Section 5 proposes and describes an Elman training method based on SGWO. Experiments and results are discussed in Section 6. Finally, we conclude with a summary of the current work and future research efforts.

## 2. Related work

Compared with traditional optimization algorithms, optimization techniques that mimic natural phenomena have dominated the field of optimization. These are also known as metaheuristic algorithms. Metaheuristic algorithms are mainly divided into three categories: evolutionary algorithms (EA), physics-based algorithms, and swarm intelligence (SI) based algorithms [24].

EA mimics the rules of nature evolves. The genetic algorithm (GA) [25] is very popular in EA. In GA, the initial solution is randomly generated and continuously updated through crossover and mutation operations. GA will find the optimal solution by iteration finally. Under the evolution of GA algorithms, many studies have proposed new algorithms, such as differential evolution (DE) [26], covariance matrix adaptation evolution strategy (CMAES) [27], evolutionary programming (EP) [28], etc.

Physics based algorithms are inspired by the physical world, such as gravity, explosions, and so on. Among them, gravitational local search (GLS) [29], multi-verse optimization algorithm (MVO) [30], sine cosine optimization algorithm (SCA) [12], and atom search optimization algorithm (ASO) [31] are classic physics based on algorithms. In GLS, the searched individuals are viewed as objects moving in space, attracting each other through gravitational interaction. Gravity forces individuals to move towards the individual with the greatest mass, gradually approaching the optimal solution.

SI is inspired by the collective behavior and nature rules of bees or herds. SI includes moth-flame optimization algorithm (MFO) [32,33], white shark optimizer (WSO) [34], whale optimization algorithm (WOA) [17], sparrow search optimization algorithm (SSA) [35], and others. In SI, the particle swarm optimization (PSO) [13] is the most popular algorithm, which updates the location of birds to find the most food.

Grey Wolf Optimizer [20] is a recently proposed metaheuristic algorithm. GWO is widely used to solve optimization problems due to its advantages such as fewer parameters and fast convergence speed. However, GWO still has poor global search ability and is easy to fall into local extremes. Recently, there have been many studies to improve the GWO algorithm in different ways. Some studies have proposed population diversity strategies to balance initial population distribution. Some works have focused on adjusting the parameters of GWO, i.e., *A* and *C*. The other works have adjusted the location update strategies to improve GWO performance. Another aspect of related studies to this work was combining GWO algorithm with other existing metaheuristic algorithms. Although SGWO algorithm is fundamentally different from previous methods, we still need to discuss the classification of metaheuristic algorithms in detail.

Modifications the random position of the initial population can balance spatial distribution of the population. Chaotic mapping strategy and opposition learning strategy were widely used in initial population. In the chaotic mapping strategy, Luo et al. [21] have proposed tent-line coupled chaotic mapping to initialize the population, which ensured that the GWO algorithm generated diverse populations; Another improved GWO algorithm used a two-dimensional chaotic map to initialize the population [22]; Zhao et al. have generated GWO initial population through Chebyshev chaotic mapping, ensuring the diversity of the initial population and enhancing global search ability of GWO [36]; In addition, some studies have integrated chaotic maps; Xu et al. have applied integrated mapping systems (CLS) to GWO to increase its population diversity and accelerate the convergence of the algorithm [37]. Besides chaotic mapping, the pseudo-antithesis number generation method based on opposition learning strategy was used to improve the distribution of population [38]; Another

improved GWO also generate its opposition wolf by lens imaging learning strategy [39]. These population diversity strategies are successful in balancing initial population distribution and improving algorithm’s performance.

Some algorithms have improved GWO performance by modifying and adjusting parameters. Song et al. [40] proposed IGWO, which enhanced exploration by modifying linear convergence factor to nonlinear; The improved grey wolf optimizer also adjusted a nonlinear parameter of GWO based on polynomials [41], and showed accurate measurement results in the optimization of seepage parameters; However, these nonlinear strategies have only succeeded in improving the performance of GWO in some aspects. For example, improved GWO [42] was beneficial to improve the convergence performance of unimodal functions, but has a poor effect on multimodal functions. Besides parameter update equations, fuzzy method [43] was used for the adaptive adjustment of the control parameters. The exploration-enhanced grey wolf optimizer (IEE-GWO) [44] used a nonlinear control parameter strategy, which has been proven that IEE-GWO has a fast convergence rate when solving unimodal functions. There are many excellent parameter adjustment strategies to improve GWO, but this method makes the algorithm perform well only on specific problems.

Some improved GWO introduced the location update strategy, making GWO suitable for a variety of optimization problems. A new search strategy named dimension learning-based hunting (DLH) [45] was introduced in IGWO, which inherited from the individual hunting behavior of wolves and shared neighboring information; An improved GWO variant used two strategies, neighbor gaze cue learning (NGCL) and random gaze cue learning [46]. These two strategies can update the location of wolves and achieve a balance between exploration and exploitation; Besides, multi-stage grey wolf optimizer (MGWO) [47] can update wolves at three stages and maintain convergence speed.

In fact, some other variants hybridize GWO with other search strategies or metaheuristic algorithms to improve its performance. Then a hybrid of genetic algorithm (GA) and GWO were combined to reduce the dimension of the obtained feature vector [48]. In another similar work, a novel improved GWO called collaboration-based hybrid GWO-SCA optimizer was developed [49]. Experimental results indicated that it was a high-performing algorithm in global optimization. With the same goal, a recently developed metaheuristic optimization algorithm called hybrid PSO-GWO [50] has been proposed to improve exploitation and exploration ability.

## 3. Grey wolf optimizer

Grey Wolf Optimizer [20] is a swarm intelligence optimization algorithm. Compared to other optimization algorithms based on population, GWO has significant differences in hunting mechanisms and mathematical models. In hunting mechanisms, GWO simulates uniquely the predation behavior according to the hierarchy of nature. The grey wolves are divided into four grades, including alpha (*α*), beta (*β*), delta (*δ*) and omega (*ω*). In groups, each of level grey wolves has a different responsibility. As a leader, *α* wolf has a powerful effect on the group and determines the hunting direction of the wolves; *β* wolf is in the second level of wolves, which helps *α* wolf in decision-making and dictates instructions to wolves in the lower hierarchy; *δ* wolf considered in third level of hierarchy, which can be following the arrangement in *α* and *β*; *ω* is at the bottom of the hierarchy. GWO hunting is abstracted as searching for optimal values. Specifically, it can be described as the following mathematical model.

### 3.1. Mathematical model for encircling the prey

The first process of hunting is encircling the prey. Eq (2) updates the position of grey wolf by calculating the distance between the grey wolf and the prey.

$$D=|C\times X_{p}(t)-X(t)|$$ (1)

$$X(t+1)=X_{p}(t)-A\times D$$ (2)

where *X*_*p*_ denotes the prey position, *X*(*t*) refers to a grey wolf position, *X*(*t*+1) represents the location of a grey wolf in the next iteration, *D* represents the distance between the grey wolf and its prey. *C* is the oscillation factor, *A* is the convergence factor. When |*A*|>1, wolves will conduct a large-scale search on the global scope. When |*A*|<1, wolves will conduct a fine search for local areas. It can be expressed by the following formula:

$$A=2ar_{2}-a$$ (3)

$$C=2r_{1}$$ (4)

a=2−2×(t/T) (5)

where *r*_1_,*r*_2_∈[0,1] is the random variable, *a* represents the distance control parameter that decreases linearly from 2 to 0, *t* is the current number of iterations and *T* is the maximum number of iterations.

### 3.2. Mathematical model for hunting mechanism

When the grey wolf tracks the prey’s position, *α* wolf will lead *β* wolf and *δ* wolf to surround the prey in nature. However, in a simulated search space we do not know the prey location. In order to build the hunting model, the optimal, sub-optimal, and third-optimal solutions are used as *α*, *β* and *δ* wolf positions. We suppose that three solutions guide other wolves to attack the prey. The position of the first three wolves will change.

$$\left\{ \begin{array}{l} D_{\alpha }=|C_{1}\times X_{\alpha }(t)-X(t)|\\ D_{\beta }=|C_{2}\times X_{\beta }(t)-X(t)|\\ D_{\delta }=|C_{3}\times X_{\delta }(t)-X(t)| \end{array}\right.$$ (6)

$$\left\{ \begin{array}{l} X_{1}=X_{\alpha }-A_{1}\times D_{\alpha }\\ X_{2}=X_{\beta }-A_{2}\times D_{\beta }\\ X_{3}=X_{\delta }-A_{3}\times D_{\delta } \end{array}\right.$$ (7)

where *X*_*α*_, *X*_*β*_, *X*_*δ*_ represent the current position of *α*, *β* and *δ*. *D*_*α*_, *D*_*β*_, *D*_*δ*_ represent the distance between the three wolves and the prey. *X*_1_, *X*_2_, *X*_3_ represent the updated position of *α*, *β* and *δ* wolf. *A*_1_, *A*_2_, *A*_3_ are defined in Eq (3), which represent respectively the convergence factor of *α*, *β* and *δ*. At this time, three wolves are the closest prey in the wolves. Therefore, individual positions are updated according to *α*, *β* and *δ* wolf position:

$$X(t+1)=\frac{X_{1}+X_{2}+X_{3}}{3}$$ (8)

The wolves continuously search for the optimal solution according to the above process. After hunting, determine *X*_*α*_ is the location of the prey.

Compared to other population-based optimization algorithms, the grey wolf optimizer has some advantages. For example, the grey wolf optimizer has a simple structure with few parameters; Grey wolf optimizer can find the optimal results quickly due to its unique hierarchy; In addition, the low time complexity of the grey wolf optimizer allows it to play an important role in practical optimization problems. However, there are still some disadvantages. For example, the grey wolf optimizer is prone to fall into local extremes. Therefore, proposing an effective improved grey wolf optimizer is one of our research objectives.

## 4. Improved grey wolf optimizer (SGWO)

To improve the optimization performance of the GWO algorithm, we proposed SGWO based on the adaptive information interaction mechanism. The SGWO algorithm was described in terms of implementation method and algorithm steps.

### 4.1. Circle population initialization (cGWO)

**Fig 1:**
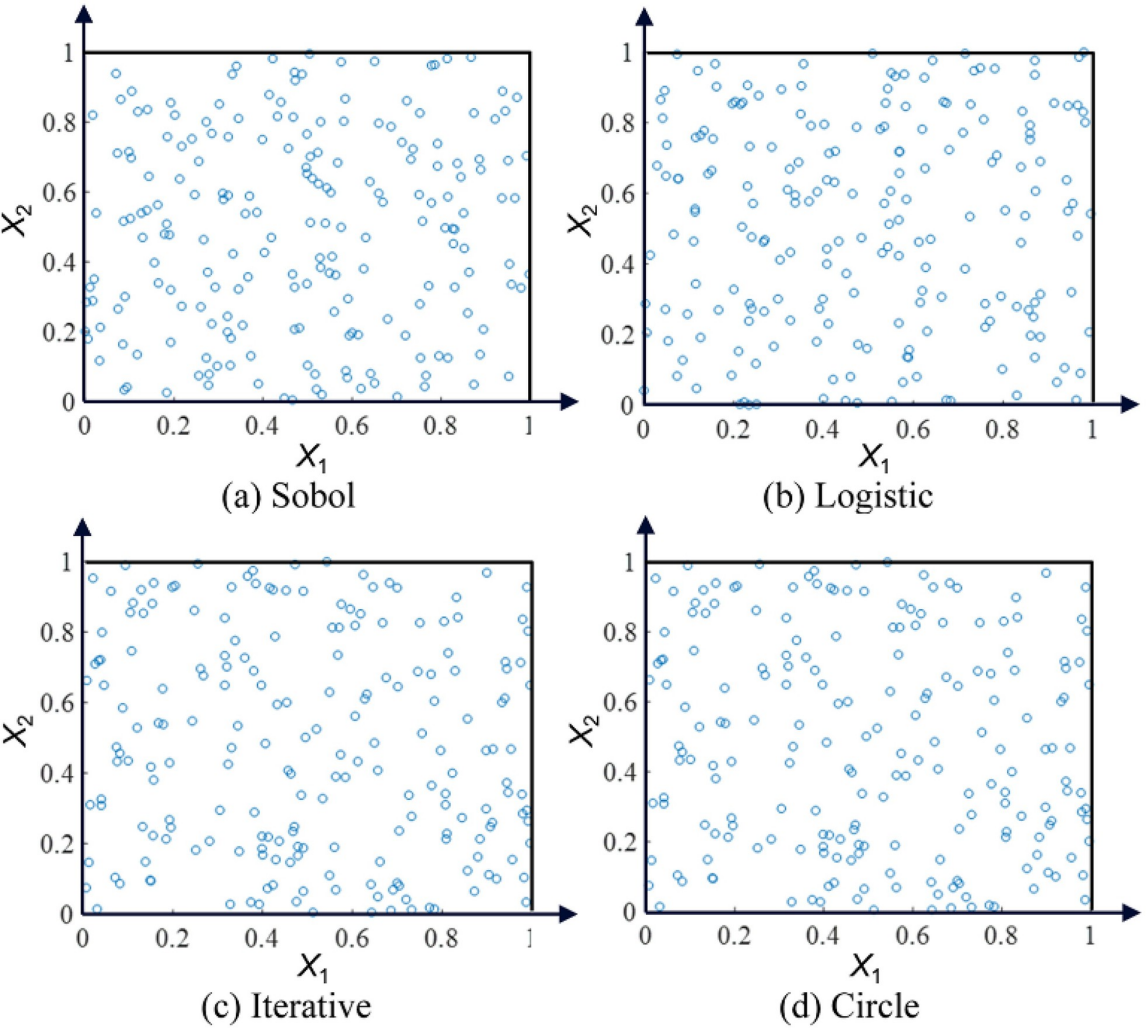
Location distribution of the four initial populations.

In GWO, the optimal value was greatly constrained by the initial position. Compared with a random search, the map was widely applied to generate the initial population because of its randomness. However, different chaotic maps have different effects. To find the optimal value quickly, we analyzed and compared Sobol, Logistic, Iterative, and Circle maps [21,22] in Fig 1.

Fig 1 gave the distribution of 200 populations at 30 iterations, respectively. For better display, the problem dimension was set to 2 dimensions. The two axis labels represented the two dimensions respectively. The search interval of the variables was set to [0, 1]. According to Fig 1, Four mapping distributions are all uniform, and the grey wolf group using Tent mapping is more evenly distributed in space than other maps. However, some individuals on the map are at the boundary, which will affect the overall efficiency of the algorithm. Compared with the four mappings, the circle map has more boundary individuals. To enhance the algorithm to deal with extreme value problems and consider experimental results, the paper still chose a circle map finally. The circle map [51] model is as follows:

$$X_{t+1}=\mathrm{mod}(X_{t}+0.2-(\frac{0.5}{2\pi })\times \sin(2\pi X_{t}),1)$$ (9)

where *X*_*t*_ represents the population individuals at the t-th iteration. The circle map is used only once in the initialization step to generate an initial population [20]. In the iteration, GWO only uses this initial population once for position updates. The circle map can balance population distribution and reverse inhibition. When the algorithm falls into a local extreme, a uniform population distribution can help wolves move to the next location. Therefore, population initialization plays a role in improving the exploration ability of SGWO.

### 4.2. Information interaction mechanism (iGWO)

In the information interaction mechanism, the hunting process was simulated as the information interaction process among wolves. Where, the hunting path as the channel, *α* position as the source point, *β* position as the transmission station, and subordinate wolves as the signal receiving point. Cauchy variation was used to change the position of source point. Golden Sine algorithm has optimized the information transmission process, and enhanced information exchange between wolves. Mathematically, the information interaction mechanism can be constructed in two steps. Every step can be explained as follows.

(1) disturbing source point

In GWO, *α* wolf position belongs to the source point, which determines the attack direction for wolves. If the leader’s position deviates, it will prolong the search time and reduce the search accuracy. Thus, the Cauchy variant [52] with excellent local exploration ability was used to optimize the head wolf. The *α* wolf can jump out of the local extreme value, and avoid premature convergence. The standard Cauchy distribution function is as follows.


$$f(x;0,1)=\frac{1}{\pi (1+x^{2})}$$ (10)

The standard Cauchy distribution function is delayed from a flat peak to both ends. A longer trailing tail can increase the perturbation probability and make the head wolf jump out of the local extremum quickly; a flat peak can reduce its search time in the adjacent area and enhance the ability to search for the global optimal solution. The standard Cauchy operator was used to randomly disturb the *α* wolf’s position. The position update formula for *α* wolf is as follows:

$$X_{1}^{'}=X_{1}+X_{1}\times Cauchy(0,1)$$ (11)

*X*_1_ is defined in Eq (7), which represents the final positions under the leadership of *α* in GWO. And *X*_1_ is calculated according to *X*_*α*_. In Eq (11), *X*_1_ is used as the initial position of *α*. $X_{1}^{'}$ is the new location of *α* wolf, which represents final position of *α* in SGWO. A Cauchy variant is helpful for *α* wolf to pass the best hunting position to wolves. Wolves can quickly close to the prey, to speed up the search speed.

(2) optimize information transmission process

In GWO, *β* wolf location belongs to the transmission station in the communication channel. However, the suboptimal value cannot determine the distance from *β* wolf to *α* wolf and *δ* wolf. Therefore, the information will be biased when *β* wolf transmits *α* wolf position to the subordinate wolves. When the algorithm is solving highly complex optimization problems, it is difficult to fully explore the solution space, which affects the search accuracy.

The golden sine algorithm (Golden-SA) [53] is a new meta-heuristic optimization algorithm. All points on the sine function are scanned by the unit circle and solution space is fully traversed. Thus, the optimal solution will be searched in Golden-SA. Updating the solution process is the core of the Golden Sine algorithm.

$$X_{i}^{i+1}=X_{i}^{t}\times |\sin(R_{1})|+R_{2}\times \sin(R_{1})\times |x_{1}\times P_{i}^{t}-x_{2}\times X_{i}^{t}|$$ (12)

where $X_{i}^{t}$ refers to a current individual position. $P_{i}^{t}$ refers to a current optimal position. *R*_1_ is [0,2*π*] random variable and *R*_2_ is [0,*π*] random variable. They control the distance and direction of movement respectively. The golden ratio *τ* is $(\sqrt{5}-1)/2$. *x*_1_ and *x*_2_ is obtained by *τ*, these two coefficients narrowing the space by spiral search and keep approaching towards the optimal solution.


$$x_{1}=-\pi +(1-\tau )\times 2\pi$$ (13)

$$x_{2}=-\pi +\tau \times 2\pi$$ (14)

Inspired by the golden section, the golden sine algorithm was incorporated into the GWO algorithm to change the movement of *β* wolf. The position update formula of the *β* wolf is as follows:

$$\left\{ \begin{array}{l} D_{\beta }^{'}=|x_{1}\times C_{2}\times X_{\beta }(t)-x_{2}\times X(t)|\\ X_{2}^{'}=|\sin(R_{1})|\times X_{\beta }+R_{2}\times |\sin(R_{1})|\times A_{2}\times D_{\beta }^{'} \end{array}\right.$$ (15)

where $D_{\beta }^{'}$ represents the new distance between the *β* wolf and the prey. $X_{2}^{'}$ represents the new position for *β* wolf in SGWO. *X*(*t*) is defined in Eq (1), which represents to a grey wolf position. *R*_1_ and *R*_2_ are defined in Eq (12), which represent random variables in [0,2*π*] and [0,*π*]. Eq (15) is updated based on Eq (7), which *A*_2_ still represents the convergence factor of *β*.

**Fig 2:**
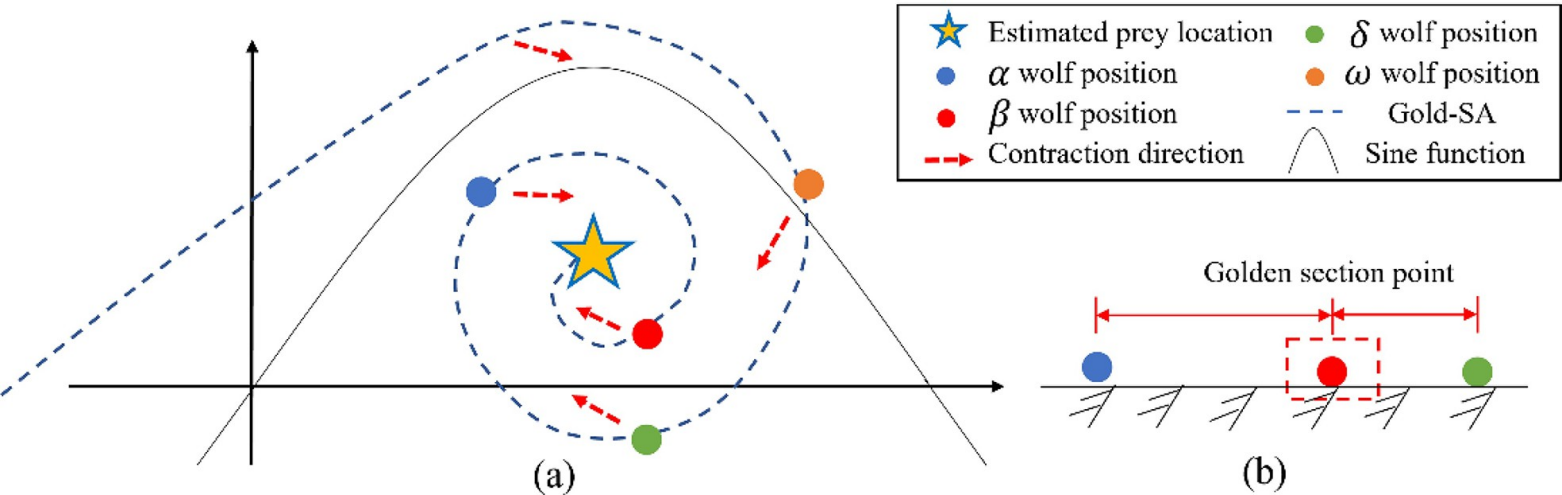
Improved the GWO principle of the golden sine algorithm.

An analysis based on Fig 2 and Eq (15) shows that: *R*_1_, *R*_2_ can constantly adjust the moving direction and moving distance of *β* wolf, so that *β* can fully understand the information difference between *α* and *δ* wolf. More specifically, *β* wolf is ensured at the golden division between *α* and *δ* wolf (as in Fig 2A). This method enhances information exchange in GWO. In addition, SGWO can scan all points on the unit circle and continuously enclose the wolves into the sine function (as in Fig 2B). Thus, wolves gradually approach the prey position (the global optimal solution), improving search speed and efficiency.

This paper transplanted the Cauchy mutation and Golden Sine algorithm as the information interaction mechanism between wolves into GWO algorithm, which can promote the information exchange between *α*, *β* and superior and subordinate wolves. The *α*, *β* wolf can release the decision results to subordinate wolves in the best transmission position. The improved SGWO can improve the shortcomings of the traditional GWO algorithm, and guide wolves to accelerate their approach to prey.

### 4.3. Adaptive position update (aGWO)

The individual position update is a key process in hunting. However, GWO always refers to the three wolf locations, making it difficult to balance global and local exploitation capability. GWO always maintains a constant update mechanism. We were inspired by the decay of the learning rate in machine learning [54], and adaptive weight *ω* was introduced at the location update. We define the *ω* in Eq (17). The updated position formula is as follows.

$$X{\left(t+1\right)}^{'}=\omega \times \frac{X_{1}^{'}+X_{2}^{'}+X_{3}}{3}$$ (16)

$$\omega (t)={\left(a+\lambda \times t\right)}^{-p},\lambda =0.99,p=0.25$$ (17)

where *a* is the distance control parameter and is defined in Eq (5). *X*_3_ represents the position of *δ* in GWO. $X_{1}^{'}$ represents the updated position of *α* by Cauchy distribution, and $X_{2}^{'}$ represents the updated position of *β* by golden sine algorithm. *X*(*t*+1)’ represents the next iteration position for a wolf, which is also the final position for a wolf in SGWO.

Due to the traditional inertia weights being artificially set, they cannot conform to the wolves hunting process. The adaptive weight factor proposed incorporated the distance control parameter so that the algorithm will adjust the search range autonomously in different periods. In the early stage of iteration, the algorithm searched the solution space globally with a large step, and in the later stage of iteration, the algorithm searched the region finely. Setting *p* to 0.25 was to avoid losing the optimal solution and reducing the accuracy of the algorithm.

**Fig 3:**
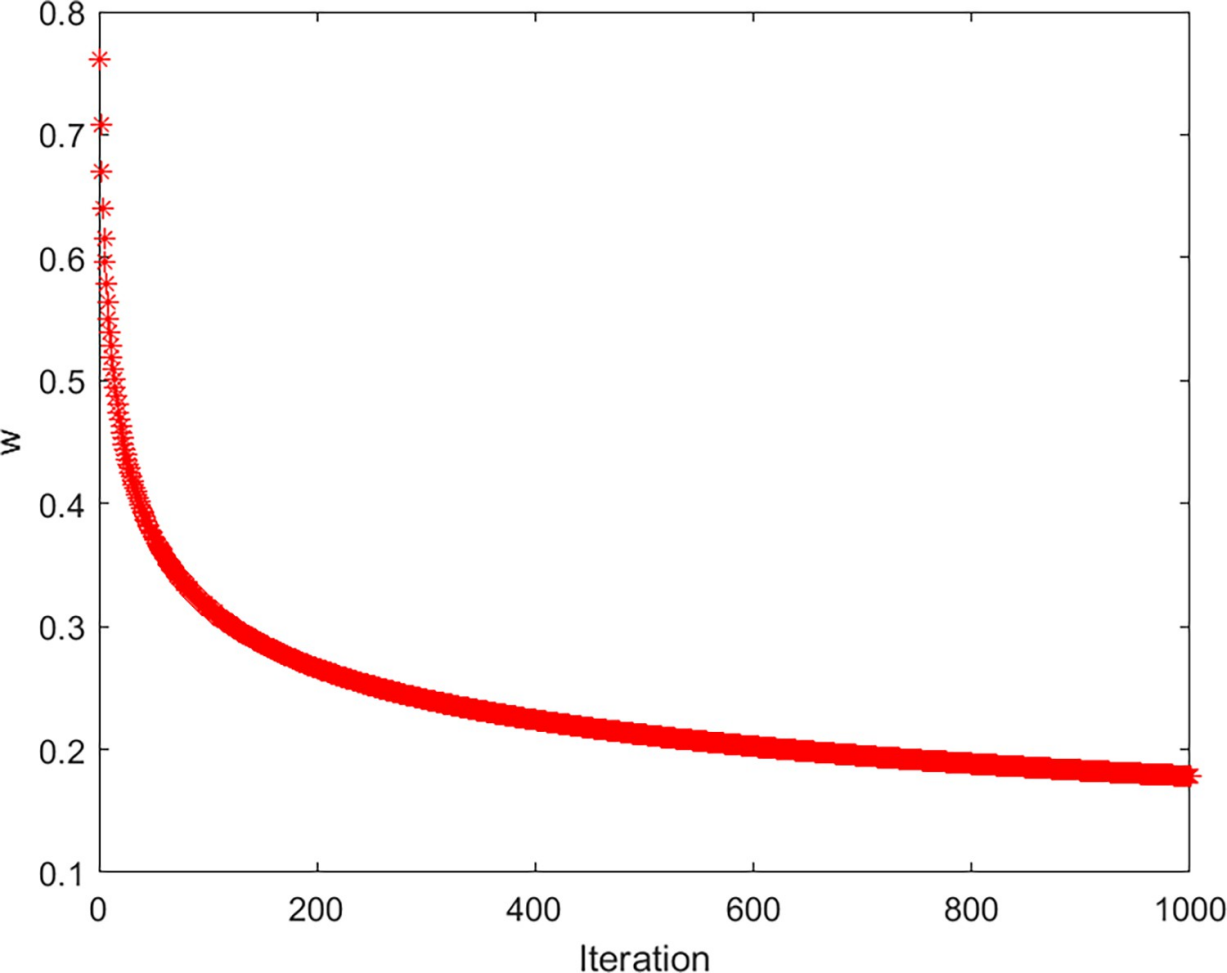
ω curve of 1000 iterations.

Form Fig 3, in the early iteration, *ω* is large for jumping out of the local extremes; in the late iteration, *ω* is smaller for improving the local search capability. Integrating adaptive weight into traditional GWO can balance global exploitation ability and local exploration ability, and find the global optimal solution quickly.

The adaptive location update mechanism is suitable for other optimization algorithms based on population, such as whale optimization algorithm (WOA) and white shark optimizer (WSO), etc. In these algorithms, this mechanism is applied to improve the formula for location update. In practice, this mechanism automatically adjusts the search step of populations by changing the parameter values, which ensures that the algorithm has global exploitation ability and local exploration ability.

### 4.4. Complexity and convergence analysis of SGWO

#### 4.4.1. Complexity analysis

The time complexity of the comparative experimental algorithm was as follows:

O(SCA)=T×n×Dim;

$$O(MVO)=T(n^{2}+n\times Dim\times \log n);$$

$$O(MFO)=T(n^{2}+n\times Dim);$$

O(WOA)=T×n×Dim;

O(GWO)=T×n×Dim;

O(SSA)=T×n×Dim;

O(ASO)=T×n×Dim;

From pseudo-code, all improved strategies are included in GWO cycle optimization process. Thus, SGWO and GWO have the same time complexity. *O*(*SGWO*) = *T*×*n*×*Dim*. Where, *T* is the maximum number of iterations, *n* is the number of populations, and *Dim* is the dimension. SGWO has few parameters, the final order is: GWO ≈ SGWO ≈ WOA ≈ SCA ≈ SSA ≈ ASO < MFO < MVO.

#### 4.4.2. Exploitation and exploration analysis

In the exploitation phase, GWO completed the hunting task by reducing the value of *a*. *a* was decreased from 2 to 0 over the course of iterations. When |*A*|>1, the wolves deviated from its prey; When |*A*|<1, the wolves attacked their prey. However, this approach led to longer exploitation times and the inability to accurately locate prey. In SGWO, we introduce an information interaction mechanism, where *β* wolf can accurately convey the position of *α* wolf to its subordinate wolves at the golden section. The wolves can quickly approach their prey through the information interaction mechanism. It is worth mentioning here that the golden sine algorithm can scan all points on a unit circle and continuously surround wolves into a sine function. Therefore, the information interaction mechanism can shorten the exploitation time of wolves. At the same time, we introduce an adaptive weight *ω* into SGWO, which can adjust the search range independently at different stages. As the *p* increases, *ω* will decrease rapidly, allowing SGWO to globally search in the solution space in larger steps. Therefore, both the information interaction mechanism and adaptive weight can improve the exploitation ability and ensure that the algorithm quickly converges to the optimal value.

In the exploration phase, GWO is prone to stagnation in local solutions. We introduce the Circle mapping and Cauchy distribution function to solve this problem. In the initial stage, circle mapping can increase population diversity, which facilitates individuals caught in extremes to find neighbors quickly. When the algorithm stalls, *α* wolf will change position by the Cauchy mutation. *α* wolf will once again lead the pack out of the stagnant region. In addition, adaptive weight *ω* also takes effect during the exploration phase. At the end of the iteration, the amplitude of *ω* decreases as the value of *λ* increases. The algorithm will search more accurately within this interval. Therefore, the adaptive weight can effectively balance the exploration and exploitation stages. SGWO also emphasizes exploitation and exploration, so as to improve the convergence speed of GWO and efficiency.

#### 4.4.3. Convergence analysis with Markov process and probability 1

Previous research has indicated that the performance of metaheuristic algorithms was improved. To date, no broad study has been performed on the theoretical analysis of metaheuristic algorithms. In this case, we have introduced innovatively Markov process and probability analysis to prove convergence performance of SGWO.

(1) convergence analysis with Markov process

**Definition 1.** Set *X* = {*X*|*X*∈*Y*} be gray wolf state space, which *x*_1_,……,*x*_*i*_∈*X*. *Y* refers to solution space and *x*_*i*_ refers to with wolf space. Set $\varphi =(X_{1},X_{2},\ldots \ldots .,X_{i}),(i=1,2,N_{\varphi })$ be wolve state. Set $X_{1},X_{2},\ldots \ldots .,X_{i}\in X$, which *X*_*i*_ refers to with wolve state set. Set $\phi =\left\{\varphi =(X_{1},X_{2},\ldots .,X_{i})|X_{i}\in X,(i=1,2,\ldots ,X_{\varphi })\right\}$ be wolves state space, which is constituted by wolve state.

**Theorem 1.** In the SGWO algorithm, Let the wolves state sequence be a finite homogeneous Markov chain and the corresponding Markov process with absorbing states.

**Proof. (1) finite homogeneous Markov chain**

Considering wolf’s state shift probabilities in the reference [55], it is known that $P(T_{\varphi }(\varphi (t-1))=\varphi (t))$ is determined by l wolf state shift probabilities. State shift probabilities are $P(T_{\varphi }(X(t-1))=X(t))$. According to Eq (15), $P(T_{\varphi }(X(t-1))=X(t))$ is related only to the state *X*(*t*−1) at the previous moment. The vector coefficients are *C*_*i*_. The *D*_*α*_, *D*_*β*_ and *D*_*δ*_ between the first three wolves and their prey. Thus, according to the definition of the Markov chain, {*φ*(*t*):*t*>0} has Markov property.

Due to search space for any optimization being finite, each *x*_*i*_ is finite. State space *X* is also finite. Because *φ* is composed of *N*_*φ*_ and *X* is a countable set, *φ* is finite. Similarly, the wolves’ state-space set *ϕ* is also finite. Therefore, {*φ*(*t*):*t*>0} is a finite Markov chain.

According to Eq (16), it is clear that *X*(*t*) is only related to the state *X*(*t*−1) at the previous moment, not the number of iterations. Thus, {*φ*(*t*):*t*>0} is a finite homogeneous Markov chain.

**Proof. (2) Markov process with absorbing states**

During each iteration, the algorithm records the current optimal top three wolf positions, so SGWO still uses an elite retention strategy. Thus, the corresponding Markov process with absorbing states.

(2) convergence analysis with probability 1

**Theorem 2.** SGWO algorithm is global convergence with probability 1.

**Proof.** To prove Theorem 2, we need to divide it into two steps. The first step is to prove that SGWO is global convergent, and then prove that the probability of convergence is 1. From the literature [56], it is clear that the conventional GWO algorithm is convergent, so that *X*(*t*+1)→*X*_*g*_(*t*) when *t*→∞. To prove the convergence of the SGWO algorithm, it is only necessary to prove that *X*(*t*+1)’→*X*_*g*_(*t*)’ when *t*→∞. That is, $\omega \times \frac{X_{1}^{'}+X_{2}^{'}+X_{3}}{3}\to 0$ when *t*→∞.

From Eq (5), *a*→0 when *t*→∞.

That is, in Eq (17), *ω*→0 when *t*→∞.

Thus, $\omega \times \frac{X_{1}^{'}+X_{2}^{'}+X_{3}}{3}\to 0$ when *t*→∞.

Therefore, SGWO is convergent.

Then SGWO satisfies the necessary and sufficient condition of global convergence in reference [55].

Thus, SGWO is the globally convergent algorithm.

Assume that at one time *t*, *X*(*t*) enters the global optimal state solution set *G*. Then at time *t*−1, *X*(*t*−1) must fall into *G*. That is P{X(t+1)∈G|X(t)∈G}=1, then

$$\begin{array}{l} P\{X(t+1)\in G\}=P\{X(t)\notin G\}\times P\{X(t+1)\in G|X(t)\notin G\}\\ \;+P\{X(t)\in G\}\times P\{X(t+1)\in G|X(t)\in G\}\\ \;=[1-P\{X(t)\in G\}]\times P\{X(t+1)\in G|X(t)\notin G\}\\ \;+P\{X(t)\in G\} \end{array}$$

Let P{X(t+1)∈G|X(t)∉G}≥v(t)≥0 and $\underset{t\to \infty }{\lim}\prod_{t=1}^{n}(1-v(t))=0$, where *v*(*t*) is the probability measure, then:

$$\begin{array}{l} 1-P\{X(t+1)\in G\}\leq [1-v(t)]\times [1-P\{X(k)\in G\}]\\ \Rightarrow 1-P\{X(t+1)\in G\}\leq [1-P\{X(k)\in G\}]\times \prod_{t=1}^{i}\left[1-v(i)\right]\\ \Rightarrow \underset{t\to \infty }{\lim}P\{X(t+1)\in G\}\geq 1 \end{array}$$

Due to P{X(t+1)∈G}≤1, $\underset{t\to \infty }{\lim}P\{X(t+1)\in G\}=1$. Thus, $\underset{t\to \infty }{\lim}P\{X(t)\in G\}=1$. We finally prove that the SGWO algorithm is a globally convergent algorithm with a probability of 1.

## 5. SGWO-Elman model construction

### 5.1. Elman neural network

**Fig 4:**
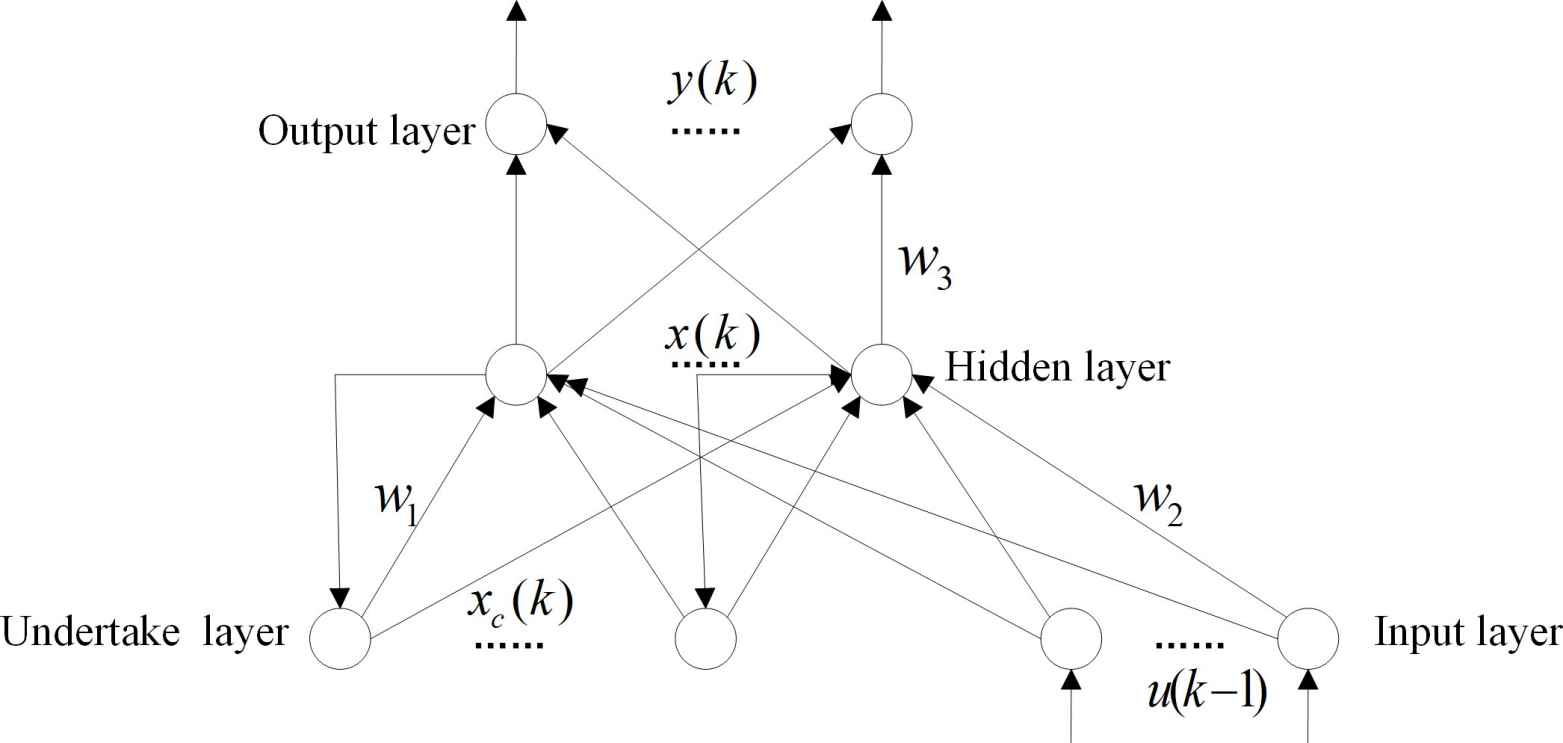
Elman neural network structure.


Elman neural network is divided into four layers: input layer, hidden layer, undertake layer, and output layer [1]. The connection of input layer, hidden layer and output layer is similar to a feedforward network. The input layer units only serve as signal transmission, while the output layer units serve as weighting. There are two types of excitation functions for hidden layer elements: linear and nonlinear. Generally, the excitation function is taken as the Sigmoid nonlinear function [2]. The receiving layer is used to remember the output value of the hidden layer unit at the previous moment, which can be considered as a delay operator with one step delay. The output of the hidden layer is used to the input of the hidden layer through the delay and storage of the undertake layer [3]. This connection method makes it sensitive to historical data. The internal feedback network improves the ability of processing dynamic information, thereby achieving dynamic modeling. The structural Elman is shown in Fig 4.

$$\left\{ \begin{array}{l} y(k)=g(\omega_{3}x(k)+b_{2})\\ x(k)=f(\omega_{1}x_{c}(k)+\omega_{2}(u(k-1))+b_{1})\\ x_{c}(k)=x(k-1) \end{array}\right.$$ (18)

The Elman model can be described as Eq (18). Where, *y* is the node vector of the output layer; *x* is the node vector of the middle layer; *u* is the input vector; *x*_*c*_ is the feedback state vector; *ω*_1_ is connection weight from hidden layer to undertake layer. *ω*_2_ is connection weight from input layer to hidden layer. *ω*_3_ is connection weight from hidden layer to output layer. *b*_1_ and *b*_2_ are the thresholds for the input layer and the hidden layer.

### 5.2. SGWO-Elman model

When Elman performs the prediction task, it first randomly selects the initial values of the parameters, then continuously updates the sample space through network training, and finally determines the best combination of parameters that fits the characteristics of the sample set. Due to the blind selection of initial parameters during the training process, the prediction effect of the network predictor is reduced and the training process is prone to fall into local extremes. Therefore, it is necessary to find the best parameters at the initial time. to train a better network structure. The optimal network parameters can better train the network structure in the iterative process. This can not only enhance the adaptability of the predictor to the dataset, but also improve the prediction accuracy. We introduced SGWO into the parameter optimization of the Elman neural network and proposed a new Elman prediction model (SGWO-Elman). This is another novel point about this paper.

The principle of the SGWO-Elman model was to replace the Elman network training problem with the weight optimization problem. Set the neural network structure is *Net*{*ω*_1_,*ω*_2_,*ω*_3_,*b*_1_,*b*_2_}. Set *X*∈[*x*_1_,*x*_2_,…..,*x*_*n*_] and $\hat{Y}\in [{\hat{y}}_{1},{\hat{y}}_{2},\ldots ..,{\hat{y}}_{n}]$ are input and output prediction sample space. Set *Y*∈[*y*_1_,*y*_2_,…..,*y*_*m*_] is the sample space to be measured. Then the search optimization objective of this paper is as follows:

$$\begin{array}{l} \min\;Net\{\omega_{1},\omega_{2},\omega_{3},b_{1},b_{2}\}\\ s.t.\left\{ \begin{array}{l} \omega_{1}\in [\omega_{1}{}_{\min},\omega_{1}{}_{\max}]\\ \omega_{2}\in [\omega_{2}{}_{\min},\omega_{2}{}_{\max}]\\ \omega_{3}\in [\omega_{3}{}_{\min},\omega_{3}{}_{\max}]\\ b_{1}\in [b_{1}{}_{\min},b_{1}{}_{\max}]\\ b_{2}\in [b_{2}{}_{\min},b_{2}{}_{\max}] \end{array}\right. \end{array}$$ (19)

This paper takes the parameter combination of the Elman neural network as training goal, the initial predictor was generated after Eq (19). The predictor was used as the gray wolf individual, to obtain the initial population. Then, the minimum mean square error (MSE) was used as the fitness function:

$$fitness=\min(MSE)=\min(\frac{1}{m}\sum_{i=1}^{m}{\left(Y-\hat{Y}\right)}^{2})$$ (20)

**Fig 5:**
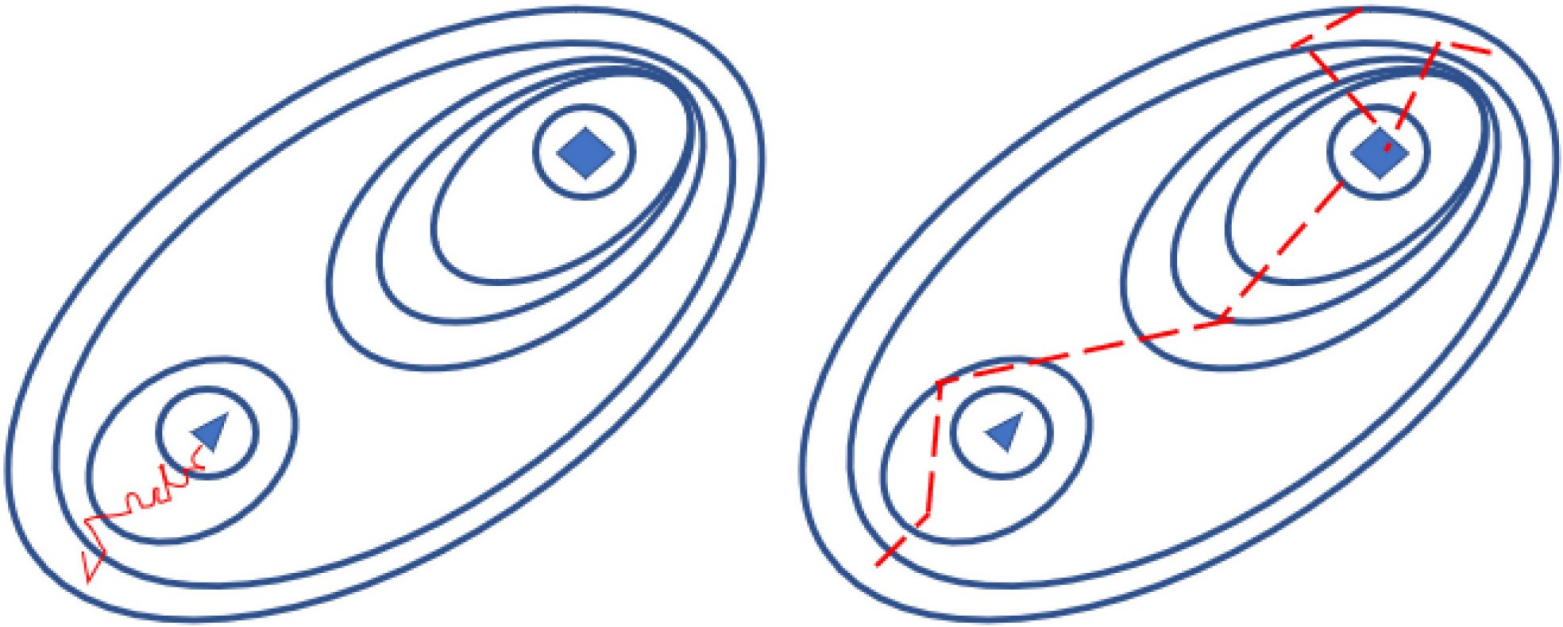
Elman and SGWO-Elman optimization process.

SGWO continuously trained the network structure through iteration. Until the optimal parameter combination was determined. Finally, the optimal network predictor can be obtained. Elman and SGWO-Elman optimization process in space is depicted in Fig 5.

In Fig 5A, Elman uses the single point search method to find the optimization route by the gradient descent, which is easy to fall into the local extremum. In Fig 5B, SGWO-Elman completes neural evolution by using the optimization algorithm, which realizes multi-point search in space. Compared with single point optimization, SGWO can find the global optimal solution. As a result of the optimization algorithm training, the network search and parameter calculation abilities have improved.

**Fig 6:**
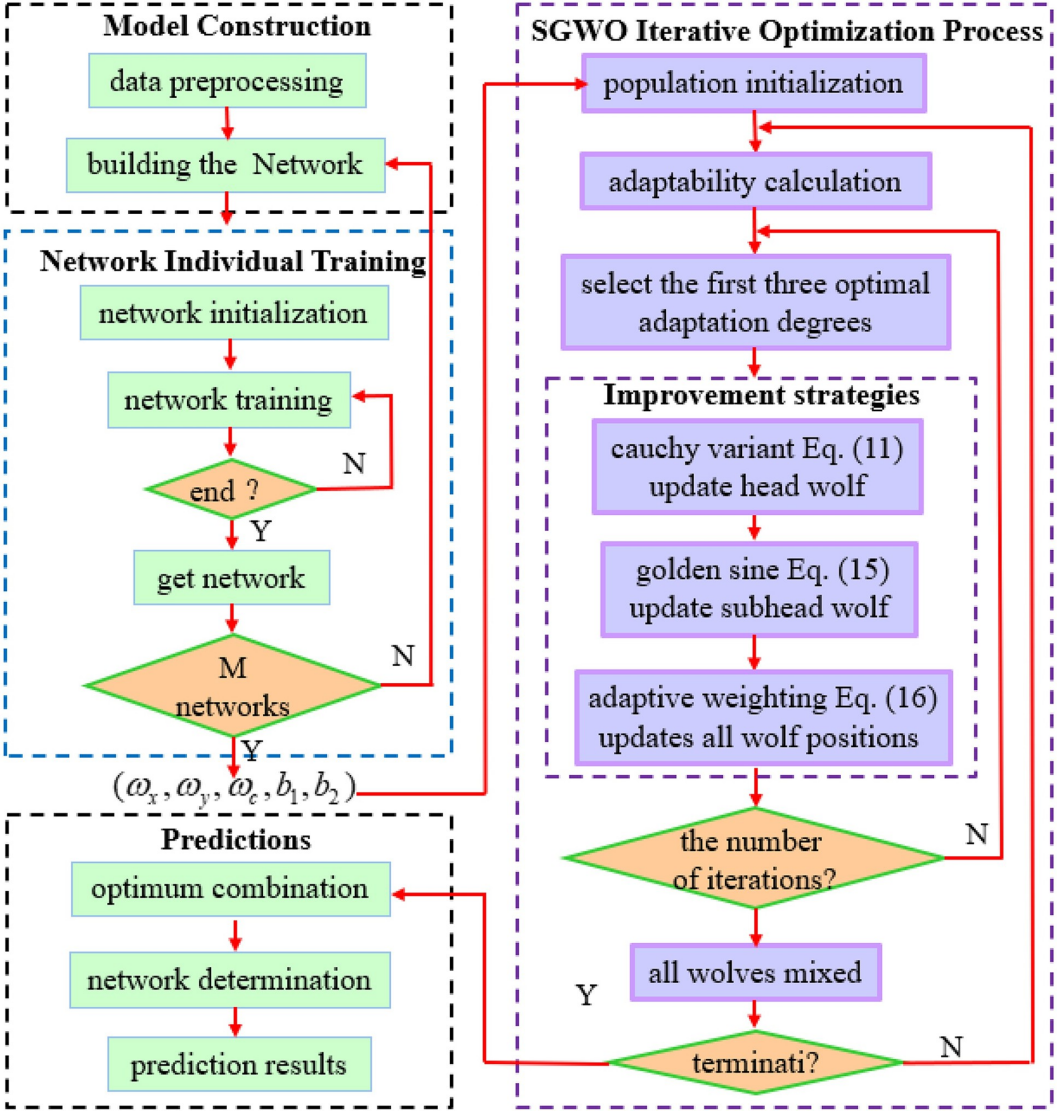
Parameter optimization of Elman neural network with SGWO for prediction.

The SGWO-Elman prediction model is specified as pseudo code and Fig 6.

**SGWO-Elman Prediction Algorithm**

input: datasets, network parameters, SGWO parameters

output: prediction results

1: Building the Elman network

2: **for** *i* = 1 to epochs **do**

3:  Training network

4: **end**

5: Get initial *Net*{*ω*_1_,*ω*_2_,*ω*_3_,*b*_1_,*b*_2_}

6: Initialization of the gray wolf population using a circle map

7: **while** (*t*<*t*_max_) **do**

8: **for** *i* = 1 to *N* **do**

9:  **for** *j* = 1 to dim **do**

10:   Calculate parameters *A* and *C* using Eqs (3) and (4)

11:   Calculate *α* and *β* locations using Eqs (11) and (15)

12:   Update individual position using Eq (16)

13:  **end for**

14: **end for**

15:Calculate individual fitness values using Eq (20)

16: Update *X*_*α*_,*X*_*β*_,*X*_*δ*_ locations

17: **end for**

18: Get the optimal *Net*{*ω*_1_,*ω*_2_,*ω*_3_,*b*_1_,*b*_2_}

19: SGWO-Elman prediction

20: Get prediction results

## 6. Results and discussion

### 6.1. SGWO comparative experiment

#### 6.1.1. Experimental information

(1) comparison methods

To ensure the experimental objective fairness, SGWO was compared with SCA, MFO, WOA, GWO, the latest variant of GWO (mGWO) [57], white shark optimizer (WSO) [34] and covariance matrix adaptation evolution strategy (CMAES) [27]. The performance of every algorithm was investigated on the 8 benchmark functions. Table 1 shows the details of 8 benchmark functions.

**Table 1:** Benchmark functions.

*f*	Function	Type	Range	*f*_min_
*f*_1_	$f_{1}(x)=\sum_{i=1}^{n}x_{i}^{2}$	Unimodal	[–100,100]	0
*f*_2_	$f_{2}(x)=\sum_{i=1}^{n}\left|x_{i}\right|+\prod_{i=1}^{n}\left|x_{i}\right|$	Unimodal	[–10,10]	0
*f*_3_	$f_{3}(x)=\sum_{i=1}^{n}{\left(\sum_{j-1}^{i}x_{j}\right)}^{2}$	Unimodal	[–100,100]	0
*f*_4_	$f_{4}(x)=\max_{i}\{|x_{i}|,1\leq i\leq n\}$	Unimodal	[–100,100]	0
*f*_5_	$\begin{array}{l} f_{5}(x)=\frac{\pi }{n}\{\left\{10\sin(\pi y_{1})+\sum_{i=1}^{n-1}{\left(y_{1}-1\right)}^{2}[1+10\sin^{2}(\pi y_{i+1})]\right\}\\ +\frac{\pi }{n}{\left(y_{n}-1\right)}^{2}+\sum_{i=1}^{n}u(x_{i},10,100,4)\\ y_{i}=1+\frac{x_{i}+1}{4}\;u(x_{i},a,k,m)=\left\{ \begin{array}{l} k{\left(x_{i}-a\right)}^{m}\;x_{i}>a\\ 0\;-a<x_{i}<a\\ k{\left(-x_{i}-a\right)}^{m}\;x_{i}<-a \end{array}\right. \end{array}$	Multimodal	[–50,50]	0
*f*_6_	$f_{6}(x)=\sum_{i=1}^{n-1}[x_{i}^{2}-10\cos(2\pi x_{i})+10]$	Multimodal	[-5.12,5.12]	0
*f*_7_	$f_{7}(x)=-20\exp(-0.2\sqrt{\frac{1}{n}\sum_{i=1}^{n}x_{i}^{2}})-\exp(\frac{1}{n}\sum_{i=1}^{n}\cos(2\pi x_{i}))+20+e$	Multimodal	[–32,32]	0
*f*_8_	$f_{8}(x)=\frac{1}{4000}\sum_{i=1}^{n}x_{i}^{2}-\prod_{i=1}^{n}\cos(\frac{x_{i}}{\sqrt{i}})+1$	Multimodal	[–600,600]	0

(2) evaluation criteria

The initialization parameter of all algorithms was same, where the population size is 50 and maximum the number of iterations is 1000. For performance testing, 30 runs have been performed in 50 dim, 100 dim and 500 dim, respectively. And experimental results were presented in terms of:

- Best of 30 runs
- Worst of 30 runs
- Mean of 30 runs
- Standard deviation of 30 runs
- Non-parametrical statistical tests
- Wilcoxon test and ranking.

#### 6.1.2. Exploitation analysis

Tables 2–4 show the results for SCA, MFO, WOA, GWO, mGWO [57], WSO [34], CMAES and SGWO in 50 dim, 100 dim, and 500 dim, respectively. From Tables 2–4, there are several conclusions can be obtained.

**Table 2:** Comparison of experimental results in 50 dim.

*f*	Index	SCA	MFO	WOA	GWO	mGWO	CMAES	WSO	SGWO
*f*_1_	mean	0.32e+02	6.67e+03	9.59e-170	9.87e-52	4.03e-65	4.06e-01	0.20e+02	**0**
	max	2.38e+02	4.00e+04	2.84e-168	3.60e-51	7.78e-65	7.18e-01	0.24e+02	**0**
	min	1.24e-01	5.86e+01	7.23e-186	1.88e-53	2.87e-66	2.34e-01	0.16e+02	**0**
	std	0.62e+02	9.58e+03	0	1.03e-51	5.29e-65	1.11e-01	0.57e+01	**0**
*f*_2_	mean	6.60e-03	0.62e+02	4.54e-108	1.45e-30	1.49e-38	0.46e+01	0.17e+01	**0**
	max	6.81e-02	1.20e+02	1.21e-106	8.21e-30	2.52e-38	0.58e+01	0.19e+01	**0**
	min	5.70e-05	0.10e+02	7.56e-118	3.33e-31	4.74e-39	0.32e+01	0.15e+01	**0**
	std	1.42e-02	0.26e+02	2.21e-107	1.41e-30	1.44e-38	6.20e-01	3.05e-01	**0**
*f*_3_	mean	2.47e+04	5.29e+04	8.56e+04	1.15e-08	1.07e-10	1.89e-03	1.18e+03	**0**
	max	5.28e+04	1.23e+05	1.42e+05	3.04e-07	2.15e-10	1.33e-02	1.38e+03	**0**
	min	5.01e+03	1.17e+04	2.00e+04	5.44e-13	1.71e-15	5.82e-07	9.75e+02	**0**
	std	1.17e+04	3.18e+04	2.14e+04	5.56e-08	1.53e-10	3.32e-02	2.92e+02	**0**
*f*_4_	mean	0.55e+02	0.80e+02	0.57e+02	3.73e-11	3.47e-15	6.30e-01	0.89e+01	**0**
	max	0.67e+02	0.88e+02	0.92e+02	2.97e-10	5.86e-15	8.99e-01	0.91e+01	**0**
	min	0.28e+02	0.75e+02	9.30e-03	7.92e-13	1.07e-15	4.38e-01	0.88e+01	**0**
	std	0.91e+01	0.38e+01	0.30e+02	5.76e-11	3.38e-15	1.13e-01	2.67e+01	**0**
*f*_5_	mean	9.73e-04	1.46e-03	1.27e-03	1.03e-02	3.07e-04	8.98e-01	3.07e-04	**2.23e-04**
	max	1.23e-03	1.55e-03	1.32e-03	2.03e-02	3.23e-04	9.12e-01	3.20e-04	**2.34e-04**
	min	6.80e-04	1.48e-03	1.22e-03	3.03e-03	3.12e-04	8.24e-01	3.12e-04	**2.09e-04**
	std	4.23e-04	3.06e-04	6.75e-04	5.42e-02	5.34e-02	1.93e-02	3.21e-04	**2.01e-04**
*f*_6_	mean	0.63e+02	2.89e+02	0	1.55e-01	0	1.74e-01	0.25e+02	**0**
	max	2.10e+02	3.64e+02	0	0.46e-01	0	2.63e-01	0.27e+02	**0**
	min	1.43e-02	2.25e+02	0	0	0	1.13e-01	0.23e+02	**0**
	std	0.51e+02	0.39e+02	0	8.52e-01	0	3.99e-02	0.30e+01	**0**
*f*_7_	mean	0.17e+02	0.18e+02	3.73e-15	2.66e-14	1.33e-14	1.84e-01	0.39e+01	**8.88e-16**
	max	0.20e+02	0.19e+02	7.99e-15	3.29e-14	1.50e-14	2.29e-01	0.43e+01	**8.88e-16**
	min	9.05e-02	6.41e-01	8.88e-16	2.22e-14	1.15e-14	1.28e-01	0.35e+01	**8.88e-16**
	std	0.65e+01	0.44e+01	2.70e-15	3.58e-15	2.51e-15	2.96e-02	5.74e+01	**0**
*f*_8_	mean	0.11e+01	0.82e+02	0	2.20e-03	0	2.07e-01	0.11e+01	**0**
	max	0.22e+01	3.61e+02	0	2.10e-02	0	3.01e-01	0.12e+01	**0**
	min	9.15e-02	4.85e-01	0	0	0	1.19e-01	0.11e+01	**0**
	std	3.89e-01	0.98e+02	0	5.80e-03	0	4.87e-02	2.40e+02	**0**

**Table 3:** Comparison of experimental results in 100 dim.

*f*	Index	SCA	MFO	WOA	GWO	mGWO	CMAES	WSO	SGWO
*f*_1_	mean	3.56e+03	2.65e+04	1.51e-169	2.04e-34	2.78e-42	0.57e+01	4.21e+02	**0**
	max	8.03e+03	5.33e+04	3.00e-168	6.37e-34	3.19e-42	0.79e+01	4.25e+04	**0**
	min	6.21e+02	3.63e+03	1.22e-185	2.27e-35	2.37e-42	0.39e+01	4.16e+04	**0**
	std	2.21e+03	1.16e+04	0	1.79e-34	5.76e-43	9.07e-01	0.64e-01	**0**
*f*_2_	mean	0.91e+02	1.76e+02	1.49e-106	7.02e-21	2.73e-26	0.21e+02	0.11e+02	**0**
	max	0.47e+01	2.93e+02	4.00e-105	1.57e-20	4.21e-26	0.25e+02	0.11e+02	**0**
	min	2.51e+02	1.03e+02	3.68e-116	2.37e-21	1.25e-26	0.18e+02	0.94e+01	**0**
	std	0.11e+01	0.54e+02	7.31e-106	3.01e-21	2.09e-26	0.17e+02	0.15e+01	**0**
*f*_3_	mean	1.83e+05	1.65e+05	6.80e+05	0.16e-01	2.00e-02	9.31e+04	4.96e+03	**0**
	max	2.96e+05	3.05e+05	8.90e+05	0.40e-02	3.99e-02	9.66e+03	5.63e+03	**0**
	min	1.09e+05	7.62e+04	4.64e+05	2.88e-05	1.82e-04	4.04e+10	4.29e+03	**0**
	std	4.54e+04	5.94e+04	1.10e+05	0.74e-01	2.81e-02	1.82e+03	9.48e+02	**0**
*f*_4_	mean	0.84e+02	0.92e+02	0.70e+02	1.08e-04	5.24e-06	0.12e+01	0.13e+02	**0**
	max	0.91e+02	0.95e+02	0.95e+02	5.47e-04	9.20e-06	0.16e+01	0.14e+02	**0**
	min	0.77e+02	0.89e+02	0.12e+02	9.99e-07	1.27e-06	0.11e+01	0.13e+02	**0**
	std	0.37e+01	0.17e+01	0.23e+02	1.56e-04	5.61e-06	1.33e+01	8.51e+01	**0**
*f*_5_	mean	6.82e-04	7.08e-04	7.71e-04	1.03e-02	1.03e-02	9.53e-01	5.07e-04	**4.83e-04**
	max	7.09e-04	7.10e-04	6.22e-03	2.03e-02	2.03e-02	9.70e-01	5.07e-04	**5.32e-04**
	min	6.56e-04	7.05e-04	5.13e-04	5.07e-04	5.07e-04	9.36e-01	5.03e-04	**4.34e-04**
	std	3.75e-05	4.17e-05	6.48e-04	1.41e-02	1.41e-02	2.37e-02	3.03e-04	**2.91e-05**
*f*_6_	mean	1.75e+02	7.06e+02	0	5.32e-01	0	9.41e-01	1.31e+02	**0**
	max	4.33e+02	8.94e+02	0	0.50e+01	0	9.77e-01	1.49e+02	**0**
	min	0.21e+02	5.84e+02	0	0	0	8.54e-01	1.12e+02	**0**
	std	1.09e+02	0.61e+02	0	0.14e-01	0	3.02e-02	0.25e+02	**0**
*f*_7_	mean	0.18e+02	0.19e+02	3.85e-15	6.87e-14	3.10e-14	9.45e-01	0.58e+01	**8.88e-16**
	max	0.21e+02	0.19e+02	7.99e-15	7.90e-14	3.99e-14	9.86e-01	0.59e+01	**8.88e-16**
	min	0.63e+01	0.18e+02	8.88e-16	5.77e-14	2.22e-14	8.80e-01	0.56e+01	**8.88e-16**
	std	0.51e+01	2.97e-01	1.89e-15	5.05e-15	1.25e-14	2.90e-02	1.89e+01	**0**
*f*_8_	mean	0.30e+02	2.20e+02	0	1.10e-03	0	9.47e-01	0.47e+01	**0**
	max	1.04e+02	5.50e+02	0	1.51e-02	0	9.81e-01	0.48e+01	**0**
	min	0.19e+01	0.36e-02	0	0	0	8.79e-01	0.46e+01	**0**
	std	0.27e+02	1.07e+02	0	3.50e-03	0	2.62e-02	1.54e+01	**0**

**Table 4:** Comparison of experimental results in 500 dim.

*f*	Index	SCA	MFO	WOA	GWO	mGWO	CMAES	WSO	SGWO
*f*_1_	mean	1.46e+05	9.19e+05	7.9e-167	5.46e-14	2.84e-17	9.40e+01	1.46e+04	**0**
	max	1.86e+05	9.51e+05	1.5e-166	7.35e-14	4.47e-17	9.58e+01	1.56e+04	**0**
	min	1.06e+05	8.88e+05	1.7e-168	3.57e-14	1.20e-17	9.21e+01	1.36e+04	**0**
	std	5.62e+04	4.46e+04	0	2.67e-14	2.31e-17	2.54e+02	1.44e+03	**0**
*f*_2_	mean	0.55e+02	2.21e+04	2.9e-102	6.79e-09	6.72e-11	8.93e+01	1.96e+02	**0**
	max	0.65e+02	2.22e+04	5.8e-102	7.05e-09	7.63e-11	9.51e+01	2.01e+02	**0**
	min	0.45e+02	2.20e+04	2.5e-112	6.53e-09	5.82e-11	8.34e+01	1.93e+02	**0**
	std	0.13e+02	0.13e+02	4.1e-102	3.67e-10	1.27e-11	8.26e+02	0.54e+01	**0**
*f*_3_	mean	3.78e+06	3.59e+06	2.26e+07	1.29e+05	5.01e+04	9.59e+01	1.46e+05	**0**
	max	3.80e+06	4.63e+06	2.28e+07	1.84e+05	6.64e+04	9.75e+01	1.53e+05	**0**
	min	3.76e+06	2.55e+06	2.23e+07	7.46e+05	3.38e+04	9.42e+01	1.39e+05	**0**
	std	3.01e+04	1.47e+06	4.16e+05	7.75e+04	2.30e+04	2.31e+02	0.97e+04	**0**
*f*_4_	mean	0.98e+02	0.98e+02	0.75e+02	0.48e+02	0.57e+02	9.50e+01	0.21e+02	**0**
	max	0.99e+02	0.98e+02	0.95e+02	0.53e+02	0.59e+02	9.53e+01	0.22e+02	**0**
	min	0.98e+02	0.98e+02	0.55e+02	0.42e+02	0.55e+02	9.47e+01	0.21e+02	**0**
	std	6.87e-01	2.27e-01	0.28e+02	0.72e+01	0.26e+01	4.18e+03	9.41e+01	**0**
*f*_5_	mean	9.72e-04	7.52e-04	5.32e-04	3.07e-04	3.05e-04	9.55e-01	3.17e-04	**2.13e-04**
	max	1.22e-03	7.82e-04	7.57e-04	3.07e-04	3.06e-04	9.69e-01	3.53e-04	**2.56e-04**
	min	7.19e-04	7.22e-04	3.07e-04	3.07e-04	3.05e-04	9.41e-01	3.47e-04	**2.24e-04**
	std	3.57e-04	4.24e-05	3.17e-04	2.44e-08	1.09e-08	1.96e-02	5.42e-02	**9.61e-05**
*f*_6_	mean	0.84e+03	6.11e+03	0	0.12e-01	5.00e-12	9.36e+01	2.24e+03	**0**
	max	1.15e+03	6.28e+03	0	0.24e-01	5.45e-12	9.65e+01	2.26e+03	**0**
	min	0.53e+03	5.93e+03	0	0.31e-01	4.54e-12	9.06e+01	2.22e+03	**0**
	std	0.44e+03	0.24e+03	0	0.17e-01	6.43e-13	4.19e-02	0.28e+02	**0**
*f*_7_	mean	0.21e+02	20.1755	8.88e-16	9.74e-09	2.91e-10	9.40e+01	0.81e+01	**8.88e-16**
	max	0.21e+02	0.21e+02	8.88e-16	1.11e-08	3.79e-10	9.51e+01	0.83e+01	**8.88e-16**
	min	0.21e+02	0.21e+02	8.88e-16	8.29e-09	2.03e-10	9.29e+01	0.77e+01	**8.88e-16**
	std	0.96e-02	0.61e-01	0	2.03e-09	1.24e-10	1.51e+02	3.68e+01	**0**
*f*_8_	mean	1.78e+03	8.17e+03	0	1.05e-14	3.88e-16	9.58e+02	1.47e+02	**0**
	max	1.88e+03	8.25e+03	0	1.52e-14	4.44e-16	9.64e+02	1.55e+02	**0**
	min	1.67e+03	8.08e+03	0	5.88e-15	3.33e-16	9.52e+02	1.39e+02	**0**
	std	0.14e+03	0.11e+03	0	6.59e-15	7.85e-17	0.89e+02	0.11e+02	**0**

1. It may be noted that the unimodal functions are suitable for testing the exploitation performance of algorithms. In unimodal functions (*f* 1—*f*_4_), all results of SGWO reach the theoretical optimum in all dimensions. At the same time, the optimization results of SGWO in three dimensions are higher than other comparison algorithms. This indicates that the SGWO has better global exploitation capability in the unimodal function. Therefore, SGWO has better stability than the other 7 algorithms.
2. From comparison algorithms, the accuracy of SGWO, GWO, mGWO and WOA is higher than other algorithms. In particular, the results of MFO in single-peak function obviously deviate from the theoretical optimal value. Although WOA has a good comprehensive effect on most functions, the optimization effect of *f*_3_ also deviates. Although mGWO and SGWO have similar search results on some functions, there is still a gap in the *f*_3_ and *f*_4_ functions. In addition, advanced WSO performs poorly on 7 functions in different dimensions. Compared to advanced metaheuristic algorithms, SGWO still outperforms WSO in all functions and dimensions. Therefore, SGWO has more advantages in exploitation ability.
3. SGWO achieves the best results in all experiments in different dimensions. Compared to other algorithms, their results show a significant decrease with the increase in dimensionality. However, SGWO is not susceptible to increased dimensions. In 500 dim, SGWO still converges to the theoretical optimal value on *f* 1—*f*_4_, *f*_6_ and *f*_8_. On *f*_5_, the results of SGWO at 500 dimensions are better than those at 50 and 100 dimensions. On *f*_7_, the results of SGWO are the same in the three dimensions. That proves that SGWO not only has prominent advantages in low dimensions, but also exhibits the best experimental results in 500 dimensions. Thus, SGWO is more suitable for solving high-dimensional problems and has a high dimensional extension.

**Fig 7:**
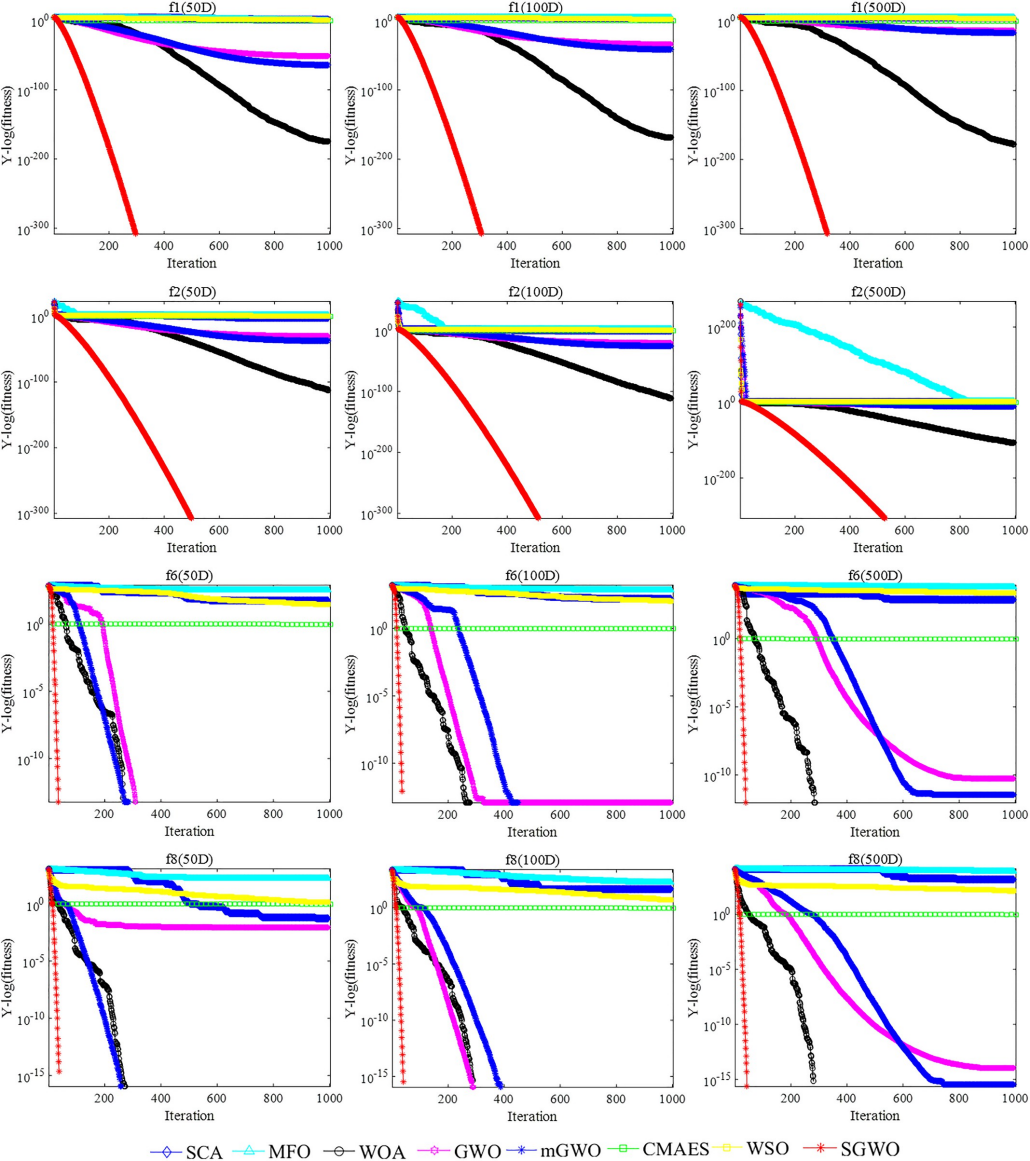
The comparison of the convergence curves.

To better compare the convergence speed of different algorithms, the convergence curves of two unimodal functions (*f*_1_, *f*_2_) and two multimodal functions (*f*_6_, *f*_8_) were analyzed in Fig 7.

1. From unimodal functions, SGWO needs 300 iterations in *f*_1_ function to converge to the theoretical optimal value, and 500 iterations in *f*_2_ function. GWO has not reached the theoretical optimal value after 1000 iterations. The optimization results of other algorithms, including mGWO and WSO, did not change significantly. This further demonstrates that SGWO can improve global exploitation capabilities.
2. With the increase of dimension, other algorithms change obviously, while SGWO ensures better convergence speed and accuracy. Thus, SGWO has significant advantages in global exploitation ability.

#### 6.1.3. Exploration analysis

Compared to unimodal functions, multimodal functions have many local optimizations, which makes them more suitable for testing the exploration capabilities of algorithms.

1. For *f*_6_ and *f*_8_, SGWO still converges to the optimal value in different dimensions. For other algorithms such as WSO and SCA, they perform poorly on these two functions. With dimensions increasing, the results of other algorithms gradually decrease. Thus, SGWO is able to provide very competitive results on *f*_6_ and *f*_8_. This indicates that SGWO has a strong advantage in jumping out of the local extreme value and SGWO has better local exploration capability.
2. For *f*_7_, neither GWO nor SGWO undergoes significant progress than other algorithms. It shows that most meta heuristic algorithms are not applicable to the optimization on *f*_7_. SGWO experimental results are still slightly higher than other algorithms on *f*_7_. Therefore, the own defects of GWO limit the effect of SGWO. This indicates that SGWO still exhibits excellent performance than other algorithms.
3. For *f*_5_, the results of all algorithms are not significantly different under the same dimension. However, SGWO still has advantages. This indicates that SGWO still exhibits excellent performance in complex functions. With dimensions increase, SGWO has the best results on 500 dimensions. This indicates that SGWO still has advantages in dealing with high-dimensional problems.
4. From Fig 7, the SGWO algorithm has a faster search speed in the same dimension compared to state-of-the-art WSO and mGWO. SGWO curve has fewer turning points, while other algorithms fall into local extreme points many times. Because the SGWO algorithm incorporated a hybrid strategy optimization leadership mechanism, the head wolf was prevented from falling in the local extremum through random disturbance. Therefore, SGWO has local exploration capability.
5. From multimodal functions, the convergence effect of SGWO, mGWO and WOA functions is obviously faster than other algorithms. At the end of iteration, the optimization results of other algorithms are not affected by the increase in iteration times. SGWO has an outstanding advantage over single-peaked functions, but the optimization performance and convergence speed still need to be improved.

#### 6.1.4. Non-parametrical statistical tests

A full statistical analysis of the optimizer comparison must be presented based on significant non-parametric tests. As the non-parametric test, Friedman test [58] was used to examine the overall performance of all algorithms. The null assumption in this test was that all algorithms would perform equally. The alternative hypothesis consists in the difference between more algorithms. We used Friedman test to analyze the results of Tables 2–4. Table 5 shows the results of the Friedman test.

**Table 5:** Friedman test for the results obtained at 50, 100 and 500 dimensions.

Dim	50	100	500
p-value	1.50e-06	8.89e-06	1.86e-06

From Table 5, the p-values for all 3 dimensions are smaller than 0.05. Therefore, the null hypothesis is rejected. This indicates that all algorithms are significantly different. In this case, we will use the "Nemenyi post-hoc test" [59] for adjusting the results for pairwise comparisons. The Nemenyi test requires to calculate the critical value.

$$\begin{array}{l} CD=q_{\alpha }\sqrt{\frac{k(k+1)}{6N}}\\ q_{\alpha }=3.301 \end{array}$$ (21)

where, *k* represents the number of algorithms. *N* represents the number of functions. After calculation, *CD* = 4.0429. To calculate the statistic, we rank the algorithm performance for each problem and compute the mean of each algorithm. Table 6 shows the results of mean ranks. Table 7 shows the mean ranks difference between each algorithm and SGWO.

**Table 6:** Mean ranks of each algorithm in different dimensions.

Dim	SCA	MFO	WOA	GWO	mGWO	CMAES	WSO	SGWO
50 Dim	6.0625	7.6250	3.7500	4.1250	2.4375	5.3750	5.3750	1.2500
100 Dim	6.5000	7.2500	3.6250	3.9375	3.0625	5.3750	5	1.2500
500 Dim	6.6875	7.1875	3.3125	3.7500	3	5.7500	5.1250	5.1250

**Table 7:** Mean ranks difference between each algorithm and SGWO.

Dim	SCA	MFO	WOA	GWO	mGWO	CMAES	WSO
50 Dim	4.8125	6.3750	2.5000	2.8750	1.1875	4.1250	4.1250
100 Dim	5.2500	6.0000	2.3750	2.6875	1.8125	4.1250	3.7500
500 Dim	5.4375	5.9375	2.0625	2.5000	1.7500	4.5000	3.8750

If the difference between the mean ranks exceeds CD, the hypothesis that the two algorithms have the same performance is rejected. From Table 7, SCA, MFO and CMAES are higher than CD at all dimensions. This indicates that SCA, MFO and CMAES all differed significantly from SGWO. There is no significant difference between other algorithms and SGWO.

#### 6.1.5. Wilcoxon test and ranking

The Friedman + Nemenyi test can express the overall performance and individual differences of SGWO. However, it is still necessary to evaluate the comparative results of each algorithm on different functions. We used the Wilcoxon rank sum test [60]. Table 8 shows the *p* values of SGWO and other algorithms, which are at *p* = 0.05 significance level.

**Table 8:** Wilcoxon rank sum test p-value of benchmark function.

*F*	SCA	MFO	WOA	GWO	mGWO	CMAES	WSO
Dim = 30							
*f*_1_	1.21e-12+	1.21e-12+	1.21e-12+	1.21e-12+	1.21e-12+	1.21e-12+	N/A =
*f*_2_	1.21e-12+	1.21e-12+	1.21e-12+	1.21e-12+	1.21e-12+	1.21e-12+	N/A =
*f*_3_	1.21e-12+	1.21e-12+	1.21e-12+	1.21e-12+	1.21e-12+	1.21e-12+	N/A =
*f*_4_	1.21e-12+	1.21e-12+	1.21e-12+	1.21e-12+	1.21e-12+	1.21e-12+	N/A =
*f*_5_	3.02e-11+	3.02e-11+	3.02e-11+	6.70e-11+	3.02e-11+	3.02e-11+	3.02e-11+
*f*_6_	1.21e-12+	1.21e-12+	1.21e-12+	0.33370+	0.08140+	1.21e-12+	N/A =
*f*_7_	1.21e-12+	1.21e-12+	1.21e-12+	1.21e-12+	6.29e-13+	1.21e-12+	N/A =
*f*_8_	1.21e-12+	1.21e-12+	1.21e-12+	0.16080+	0.04190+	1.21e-12+	N/A =
*+/ = /-*	8/0/0	8/0/0	8/0/0	8/0/0	8/0/0	8/0/0	1/7/0
Dim = 50							
*f*_1_	1.21e-12+	1.21e-12+	1.21e-12+	1.21e-12+	1.21e-12+	1.21e-12+	1.21e-12+
*f*_2_	1.21e-12+	1.21e-12+	1.21e-12+	1.21e-12+	1.21e-12+	1.21e-12+	1.21e-12+
*f*_3_	1.21e-12+	1.21e-12+	1.21e-12+	1.21e-12+	1.21e-12+	1.21e-12+	1.21e-12+
*f*_4_	1.21e-12+	1.21e-12+	1.21e-12+	1.21e-12+	1.21e-12+	1.21e-12+	1.21e-12+
*f*_5_	3.02e-11+	3.02e-11+	1.21e-10+	3.02e-11+	3.02e-11+	3.02e-11+	3.02e-11+
*f*_6_	1.21e-12+	1.21e-12+	N/A =	0.04190+	N/A =	1.21e-12+	1.21e-12+
*f*_7_	1.21e-12+	1.21e-12+	7.46e-07+	5.68e-13+	4.42e-13+	1.21e-12+	1.21e-12+
*f*_8_	1.21e-12+	1.21e-12+	N/A =	0.04190+	N/A =	1.21e-12+	1.21e-12+
*+/ = /-*	8/0/0	8/0/0	6/2/0	8/0/0	6/2/0	8/0/0	8/0/0
Dim = 100							
*f*_1_	1.21e-12+	1.21e-12+	1.21e-12+	1.21e-12+	1.21e-12+	1.21e-12+	1.21e-12+
*f*_2_	1.21e-12+	1.21e-12+	1.21e-12+	1.21e-12+	1.21e-12+	1.21e-12+	1.21e-12+
*f*_3_	1.21e-12+	1.21e-12+	1.21e-12+	1.21e-12+	1.21e-12+	1.21e-12+	1.21e-12+
*f*_4_	1.21e-12+	1.21e-12+	1.21e-12+	1.21e-12+	1.21e-12+	1.21e-12+	1.21e-12+
*f*_5_	3.02e-11+	3.02e-11+	6.07e-11+	3.02e-11+	3.02e-11+	3.02e-11+	3.02e-11+
*f*_6_	1.21e-12+	1.21e-12+	N/A =	1.06e-07+	N/A =	1.21e-12+	1.21e-12+
*f*_7_	1.21e-12+	1.21e-12+	2.09e-09+	8.52e-13+	7.39e-13+	1.21e-12+	1.21e-12+
*f*_8_	1.21e-12+	1.21e-12+	N/A =	0.08150+	N/A =	1.21e-12+	1.21e-12+
*+/ = /-*	8/0/0	8/0/0	6/2/0	8/0/0	6/2/0	8/0/0	8/0/0
Dim = 500							
*f*_1_	1.21e-12+	1.21e-12+	1.21e-12+	1.21e-12+	1.21e-12+	1.21e-12+	1.21e-12+
*f*_2_	1.21e-12+	1.21e-12+	1.21e-12+	1.21e-12+	1.21e-12+	1.21e-12+	1.21e-12+
*f*_3_	1.21e-12+	1.21e-12+	1.21e-12+	1.21e-12+	1.21e-12+	1.21e-12+	1.21e-12+
*f*_4_	1.21e-12+	1.21e-12+	1.21e-12+	1.21e-12+	1.21e-12+	1.21e-12+	1.21e-12+
*f*_5_	3.02e-11+	3.02e-11+	5.62e-10+	3.02e-11+	3.02e-11+	3.02e-11+	3.02e-11+
*f*_6_	1.21e-12+	1.21e-12+	N/A =	1.16e-06+	1.16e-09+	1.21e-12+	1.21e-12+
*f*_7_	1.21e-12+	1.21e-12+	3.12e-08+	6.23e-13+	7.94e-13+	1.21e-12+	1.21e-12+
*f*_8_	1.21e-12+	1.21e-12+	N/A =	0.1145+	0.3409+	1.21e-12+	1.21e-12+
*+/ = /-*	8/0/0	8/0/0	6/2/0	8/0/0	8/0/0	8/0/0	8/0/0

*Note: "+", "-", " = " indicate that SGWO advanced, worsened, and is equivalent to the comparison algorithm, and N/A indicates "not applicable".

It can be seen from Table 8 that SGWO is more statistically significant than all other algorithms except mGWO and WOA. In 50 and 100 dim, the results between mGWO and WOA are not applicable on *f*_6_ and *f*_8_. These prove that their significance with SGWO is lower. In 500 dim, the results of WOA and GWO on *f*_6_ and *f*_8_ are higher than 50 and 100 dimensions, indicating that there is a significant difference between SGWO and these two algorithms. Therefore, the SGWO algorithm is not affected by dimensions and can be extended to high dimensions. SCA, MFO, WSO and CMAES have the same result on different functions. This indicates SGWO is significantly different from SCA, MFO, WSO. However, CMAES differs less from SGWO in the overall comparison Table 7. All the above analyses are consistent with the results in Table 7. Meanwhile, all results are the same on *f* 1—*f*_4_, but the result of *f*_5_ is higher than other functions. This shows that each algorithm has a lower optimization effect on *f*_5_. With the increase of dimensions, the results have little difference in different dimensions.

In conclusion, SGWO is superior to other comparison algorithms. SGWO has significantly better optimization performance and comprehensive strength. In addition, we used MAE to sort the all algorithms [61]. MAE expression is as follows:

$$MAE=\frac{\sum_{i=1}^{N_{f}}\left|Mean_{i}-o_{i}\right|}{N_{f}}$$ (22)

where, *Mean*_*i*_ is the mean value of the algorithm. *o*_*i*_ is the theoretical optimal value of the benchmark function. *N*_*f*_ is the number of benchmark functions. Table 9 shows MAE under different dimensions. Table 10 shows the sum of MAE in each algorithm. *MAE* = (*MAE*_50_+*MAE*_100_+*MAE*_500_)/3.

**Table 9:** Algorithm ranking under different dimensions.

50 Dim			100 Dim			500 Dim		
Name	MAE	Rank	Name	MAE	Rank	Name	MAE	Rank
SGWO	1.02e-05	1	SGWO	8.75e-06	1	SGWO	2.66e-05	1
mGWO	3.84e-05	2	mGWO	3.80e-03	2	CMAES	1.90e+02	2
GWO	1.98e-02	3	GWO	2.71e-01	3	mGWO	6.27e+03	3
CMAES	8.41e-01	4	CMAES	4.25060	4	GWO	1.61e+04	4
WSO	1.55e+02	5	WSO	6.93e+02	5	WSO	2.04e+04	5
SCA	0.31e+04	6	SCA	0.23e+05	6	SCA	4.91e+05	6
MFO	0.75e+04	7	MFO	0.24e+05	7	MFO	5.68e+05	7
WOA	0.11e+05	8	WOA	0.85e+05	8	WOA	2.82e+06	8

**Table 10:** Total ranking of algorithms in different dimensions.

Name	MAE	Dim	Rank	Total ranking
SGWO	8.87e-06	50/100/500	1/1/1	1
CMAES	0.65e+02	50/100/500	4/4/2	2
mGWO	2.09e+03	50/100/500	2/2/3	3
GWO	5.37e+03	50/100/500	3/3/4	4
WSO	7.08e+03	50/100/500	5/5/5	5
SCA	1.72e+05	50/100/500	6/6/6	6
MFO	1.99e+05	50/100/500	7/7/7	7
WOA	9.72e+05	50/100/500	8/8/8	8

From Table 9, all algorithms rank differently in each dim. With the increase of dim, the MAE values change significantly for other algorithms, but SGWO can maintain the optimal level. This indicates that SGWO has strong stability and is not easily affected by dim changes. In 50 and 100 dimensions, the ranking of the eight algorithms is the same, SGWO > mGWO > GWO > CMAES > WSO > SCA > MFO >WOA respectively. CMAES ranks higher than GWO and mGWO in the 500 dimensions, which shows that the performance effect of each algorithm is different in the three dimensions. GWO is lower than SGWO in every three dimensions. This proves that the comprehensive performance of GWO is better than other comparison algorithms, and the improved strategy proposed in this paper significantly improves the optimization effect of GWO. The sorting results in Table 10 are SGWO > CMAES > mGWO > GWO > WSO > SCA > MFO > WOA, respectively.

**Fig 8:**
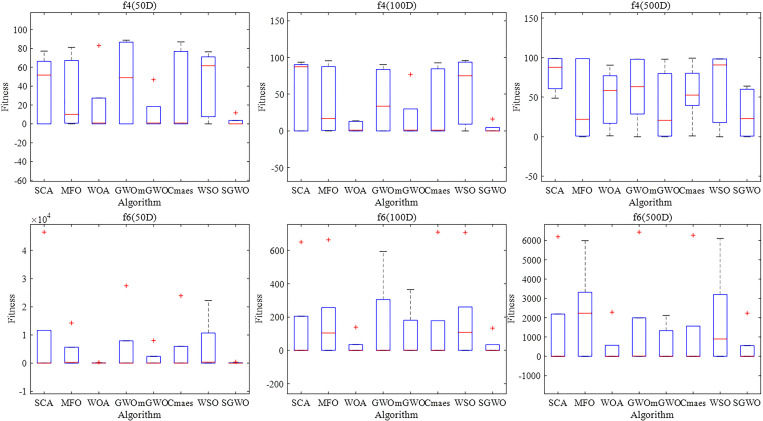
Boxplot of fitness in various algorithms.

Fig 8 shows the box convergence diagram of two benchmark functions in different dimensions. The two benchmark functions are a unimodal function *f*_4_ and multimodal functions *f*_6_ respectively.

From Fig 8, The fitness values of SGWO are lower than other comparison algorithms, even close to zero. It shows that the improved strategy based on an adaptive information interaction mechanism is effective for traditional GWO. The median of SGWO is lower than other algorithms whether in different dim or peaks. This shows that SGWO can get a better optimization effect after multiple iterations. At the same time, the interquartile spacing of SGWO is short than other algorithms, which indicates that the optimization effect of SGWO is more concentrated under each function and dimension.

### 6.2. Three strategies comparative experiment

#### 6.2.1. Experimental information

In order to analyze the impact of different strategies on the SGWO algorithm, we conducted comparative experiments on four algorithms. cGWO is the first strategy “Circle population initialization”; iGWO is the second strategy “Information interaction mechanism”; aGWO is the third strategy “Adaptive position update”; aWOA is the application of the third strategy to WOA.

To ensure the experimental objective fairness, The initialization parameter of all algorithms was same, where the population size is 50 and maximum the number of iterations is 1000. For performance testing, 30 runs have been performed in 50 dim, 100 dim and 500 dim, respectively. And experimental results are presented in terms of:

- Best of 30 runs
- Standard deviation of 30 runs.

#### 6.2.2. cGWO analysis

**Fig 9:**
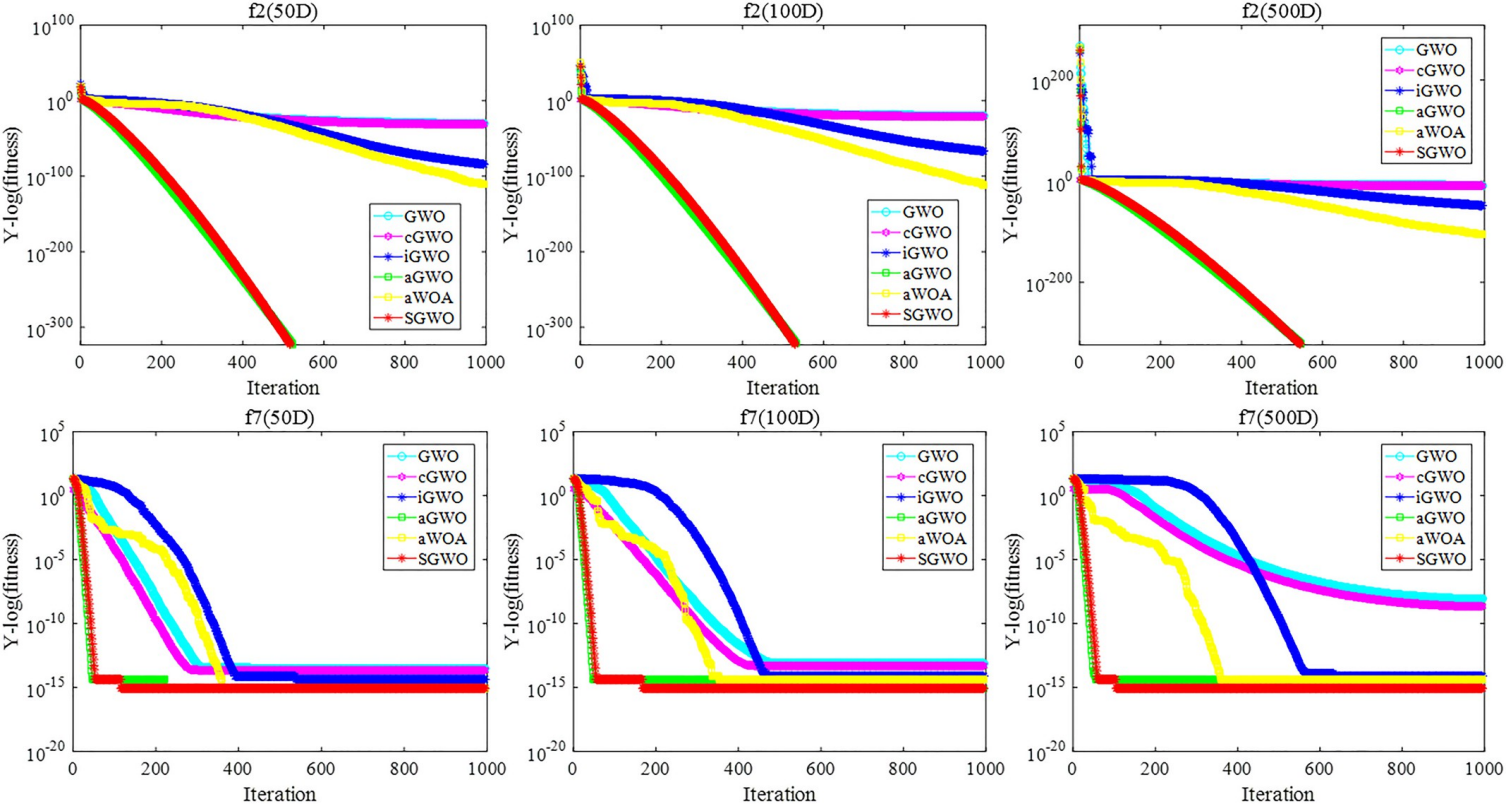
Convergence curves for different strategies.

Table 11 shows the results for GWO, cGWO, iGWO, aGWO, aWOA and SGWO in 50 dim, 100 dim, and 500 dim, respectively. Fig 9 shows the convergence curves of different algorithms in unimodal functions *f*_2_ and multimodal functions *f*_7_.

**Table 11:** Experimental results of three strategies in 50, 100 and 500 dims.

No.	Index	*f*_1_	*f*_2_	*f*_3_	*f*_4_	*f*_5_	*f*_6_	*f*_7_	*f*_8_
50Dim									
GWO	mean	9.87e-52	1.45e-30	1.15e-08	3.73e-11	1.03e-02	1.55e-01	2.66e-14	2.20e-03
	std	5.29e-65	8.21e-30	5.56e-08	5.76e-11	5.42e-02	8.52e-01	3.58e-15	5.80e-03
cGWO	mean	9.46e-55	2.66e-34	1.07e-10	1.47e-14	4.42e-03	5.68e-15	2.82e-14	1.29e-05
	std	1.11e-71	2.59e-33	2.38e-10	1.14e-13	8.40e-03	1.79e-14	2.39e-15	1.49e-05
iGWO	mean	7.74e-150	7.09e-84	3.31e-80	8.74e-54	3.09e-04	0	5.86e-15	0
	std	1.49e-149	9.45e-84	4.73e-80	1.69e-53	1.03e-06	0	1.83e-15	0
aGWO	mean	0	0	0	0	6.36e-04	0	2.30e-15	0
	std	0	0	0	0	1.50e-04	0	1.83e-15	0
aWOA	mean	4.42e-16	4.39e-107	4.97e+08	0.87e-02	3.86e+04	0.67e-01	8.88e-16	5.59e-03
	std	4.86e-16	6.21e-107	3.14e+08	0.54e-01	2.70e+04	0.94e-01	2.00e-14	7.98e-03
SGWO	mean	0	0	0	0	2.23e-04	0	8.88e-16	0
	std	0	0	0	0	0	0	0	0
100Dim									
GWO	mean	2.04e-34	7.02e-21	0.16e-01	1.08e-04	1.03e-02	5.32e-01	3.10e-14	1.10e-03
	std	1.79e-34	3.01e-21	0.74e-01	1.56e-04	1.41e-02	0.14e-01	1.25e-14	3.50e-03
cGWO	mean	3.23e-35	1.06e-26	1.27e-03	3.41e-05	2.31e-03	2.32e-15	7.05e-15	1.01e-03
	std	3.03e-35	7.90e-26	1.34e-03	3.32e-05	6.34e-03	0.10e-15	7.14e-15	3.21e-03
iGWO	mean	1.72e-119	2.88e-67	2.72e-59	6.11e-38	4.95e-04	0	7.28e-15	0
	std	4.19e-119	2.02e-67	8.62e-59	1.64e-37	3.91e-04	0	1.49e-15	0
aGWO	mean	0	0	0	0	7.98e-04	0	3.73e-15	0
	std	0	0	0	0	3.24e-04	0	1.49e-15	0
aWOA	mean	1.61e-16	9.94e-111	2.11e+09	8.49e+01	1.66e+04	5.13e-03	2.66e-15	1.49e+00
	std	2.28e-16	1.30e-110	2.91e+08	0.38e+01	9.53e+03	7.12e-02	6.76e-15	2.11e+00
SGWO	mean	0	0	0	0	4.83e-04	0	8.88e-16	0
	std	0	0	0	0	2.91e-05	0	0	0
500Dim									
GWO	mean	5.46e-14	6.79e-09	1.29e+05	0.48e+02	3.07e-04	0.12e-01	9.74e-09	1.05e-14
	std	7.35e-14	3.67e-10	7.75e+04	0.72e+01	2.44e-04	0.17e-01	2.03e-09	6.59e-15
cGWO	mean	2.60e-16	5.16e-13	1.03e+05	0.23e+01	6.73e-04	0.13e-12	8.14e-11	1.02e-16
	std	8.32e-17	1.28e-13	5.27e+04	0.37e+01	5.01e-04	0.29e-12	2.80e-11	3.90e-16
iGWO	mean	1.00e-86	1.06e-48	3.74e-42	3.14e-13	4.93e-04	0	7.99e-15	0
	std	1.83e-86	1.88e-49	5.98e-42	1.97e-13	4.10e-04	0	0	0
aGWO	mean	0	0	0	0	6.15e-04	0	4.44e-15	0
	std	0	0	0	0	1.11e-04	0	0	0
aWOA	mean	2.29e-14	5.35e-108	4.81e+07	5.42e+01	2.66e+17	1.68e-03	2.66e-15	6.17e-03
	std	3.22e-14	7.57e-108	2.66e+07	3.63e+01	5.18e+18	2.34e-03	2.65e-15	8.72e-03
SGWO	mean	0	0	0	0	2.13e-04	0	8.88e-16	0
	std	0	0	0	0	9.61e-05	0	0	0

From Table 11, compared to GWO, the results of cGWO have improved slightly in all functions of different dimensions. This indicates that the circle population initialization strategy can improve the optimization ability of GWO. However, the improvement effect of cGWO is weaker than iGWO, aGWO and SGWO. Specifically, circle population initialization was used only once in the initialization step, which weakened the effect of cGWO.

From Fig 9, in unimodal functions *f*_2_, although the convergence speed of cGWO algorithm is slightly higher than GWO, it is still not as good as other strategies. In multimodal functions *f*_7_, the convergence speed of cGWO is better than GWO and iGWO. At the same time, the number of transitions in cGWO should be less than aGWO, aWOA and SGWO. This indicates that circle population initialization can help cGWO jump out of local extremum. Therefore, cGWO can not only improve the exploration ability of GWO, but also contribute to improving SGWO.

#### 6.2.3. iGWO analysis

From Table 11, compared to GWO, the results of iGWO have improved significantly in all functions of different dimensions. In unimodal functions *f* 1—*f*_4_, iGWO can be improved dozens of times. In *f*_6_ and *f*_8_, iGWO can reach the theoretical optimal value. This indicates that the information interaction mechanism can improve the convergence ability.

From Fig 9, in unimodal functions *f*_2_, the convergence speed of iGWO is higher than GWO and cGWO. In multimodal functions *f*_7_, the convergence speed of iGWO is lower than GWO, cGWO and aWOA at the beginning of the iteration. However, the convergence speed of iGWO is higher than GWO, cGWO and aWOA at the end of the iteration. Therefore, the information interaction mechanism will contribute to generally the efficiency of SGWO.

#### 6.2.4. aGWO analysis

From Table 11, aGWO can reach the theoretical optimal value in *f* 1—*f*_4_, *f*_6_ and *f*_8_. The results of aGWO are not significantly different from SGWO. This indicates that adaptive position update strategy can improve optimization performance of GWO and play an important role in SGWO. Meanwhile, information interaction mechanism is the best strategy compared to the other two strategies. From Fig 9, the convergence speed of aGWO is the same as SGWO. And they can quickly converge to the optimal value.

On the meanwhile, we incorporate adaptive position update strategy into WOA. Although the convergence performance of aWOA is not as good as that of aGWO, it is still superior to GWO. From Table 10, it can be seen that GWO performs better than WOA. Therefore, aGWO > aWOA > GWO > WOA. The information interaction mechanism can also improve the optimization performance of WOA. This further proves that the information interaction mechanism is an effective strategy.

### 6.3. Sensitivity analysis of parameters

The sensitivity analysis of two control parameters of Eq (17) is investigated in this section. These two parameters are *λ* and *p*, which together control the change of *ω* in the iteration. On the meanwhile, *ω* plays an important role in balancing exploration and exploitation. Therefore, it is necessary to conduct sensitivity analysis on *λ* and *p*.

Table 12 represents *ω* mean by 1000 iterations under various parameter combinations. As shown in Table 12, when *p* is constant, the mean value of *ω* gradually decreases as *λ* increases. When *λ* is constant, as *p* increases, the mean value of *ω* gradually decreases and decays faster. In the seventh experiment, when *λ* and *p* reached the maximum, the mean value of *ω* was the minimum. The results can be interpreted as saying that *λ* and *p* are negatively correlated with *ω*.

**Table 12:** Sensitivity analysis of *λ* and *p*.

Number	Value of parameters		Mean(*ω*)
	*λ*	*p*	
1	0.2	0.1	0.6501
2	0.4	0.1	0.6080
3	0.6	0.1	0.5844
4	0.2	0.25	0.3465
5	0.4	0.25	0.2937
6	0.6	0.25	0.2662
7	0.99	0.25	**0.2356**

**Fig 10:**
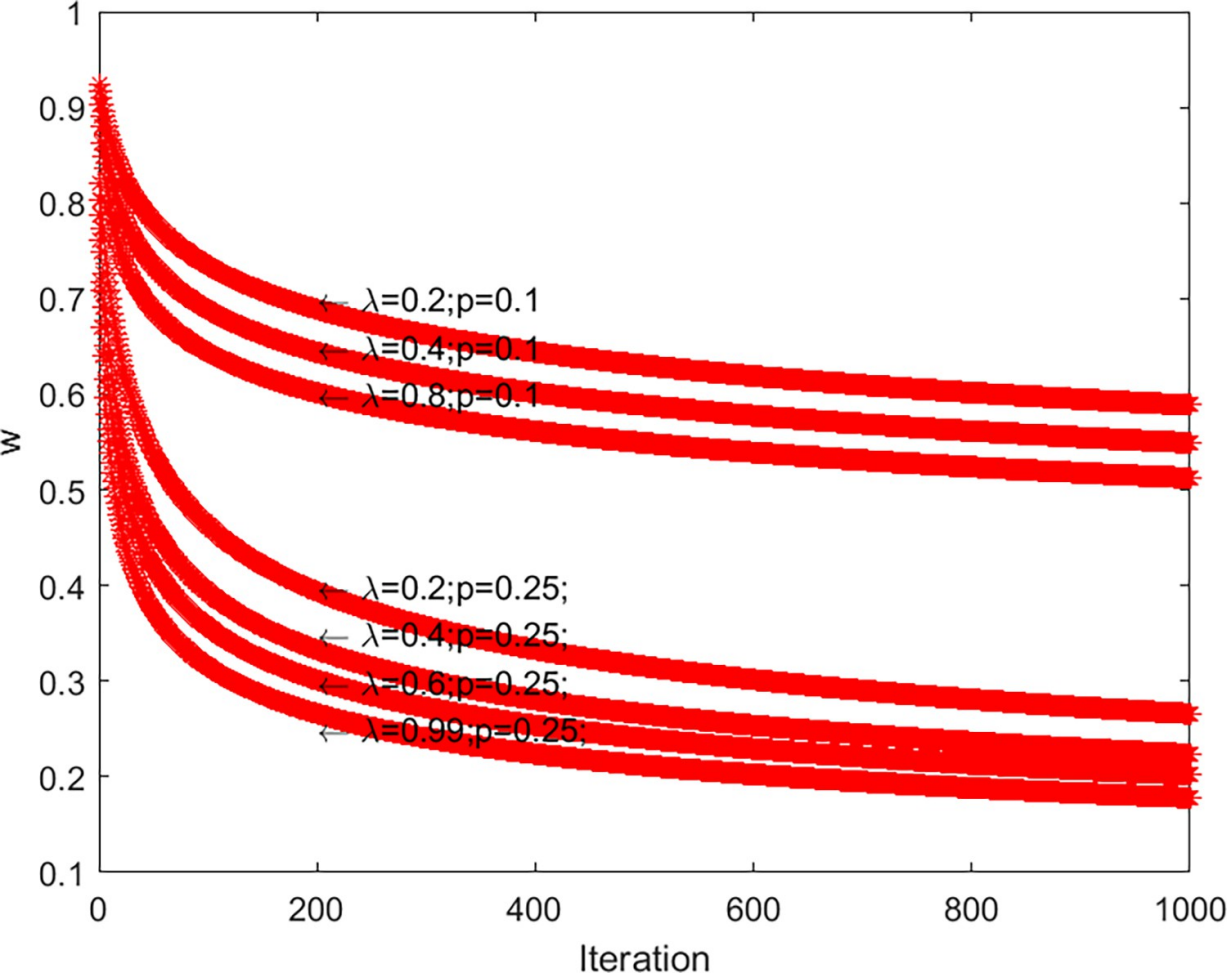
*ω* curve with different parameters.

Fig 10 represents *ω* curves of 1000 iterations under various parameter combinations. When *λ* is constant, with the increase of *p*, the value of *ω* decreases rapidly in the early stage of the iteration. That proves that *p* can exploitation time and quickly find the optimal value range for SGWO. At the end of the iteration, as the value of *λ* increases, *ω* will quickly transition to the exploration phase. With the increase of the iterations, the amplitude of the *ω* is decreased, which proves that SGWO will refine the search solution. Therefore, we set *λ* to the maximum to improve the SGWO’s exploration performance. Although increasing the *p* will accelerate the decrease in *ω*, considering that SGWO needs to balance exploration and exploitation, we set *p* to 0.25.

### 6.4. SGWO for practical applications

#### 6.4.1. SGWO for tension/compression spring design problem

The objective of this problem is to minimize the weight of a tension/compression spring [62]. This problem can be abstracted into the following mathematical model. In the model, *x*_1_ is wire diameter, *x*_2_ is mean coil diameter, and *x*_3_ is the number of active coils.


$$\begin{array}{l} consider\;\vec{x}=[x_{1},x_{2},x_{3}]\\ \min\;f(\vec{x})=(x_{3}+2)x_{2}x_{1}^{2}\\ s.t.\;g_{1}(\vec{x})=1-\frac{x_{2}^{3}x_{3}}{71785x_{1}^{4}}\leq 0\\ \;g_{2}(\vec{x})=\frac{4x_{2}^{2}-x_{1}x_{2}}{12566(x_{2}x_{1}^{3}-x_{1}^{4})}+\frac{1}{5108x_{1}^{2}}\leq 0\\ \;g_{3}(\vec{x})=1-\frac{140.45x_{1}}{x_{2}^{2}x_{3}}\leq 0\\ \;g_{4}(\vec{x})=\frac{x_{1}+x_{2}}{1.5}-1\leq 0\\ variable\;range\;0.05\leq x_{1}\leq 2.00;0.25\leq x_{2}\leq 1.30;2.00\leq x_{3}\leq 15.0 \end{array}$$ (23)

Table 13 shows the comparison of results of the tension/compression spring design problem. Table 13 suggests that SGWO finds a design with the minimum weight for this problem. This further proves that SGWO can be applied to practical problems and exhibits better performance.

**Table 13:** Tension/Compression spring design problem results in other algorithms.

Algorithm	mean	max	min	std
SCA	1.30e-02	1.31e-02	1.28e-02	1.10e-0
MFO	1.31e-02	1.35e-02	1.27e-02	3.11e-04
WOA	1.32e-02	1.36e-02	1.31e-02	1.89e-02
GWO	1.27e-02	1.27e-02	1.26e-02	2.67e-05
mGWO	1.27e-02	1.27e-02	1.26e-02	2.77e-05
CMAES	9.48e-01	9.69e-01	8.79e-01	3.89e-02
WSO	1.26e-02	1.26e-02	1.26e-02	9.92e-07
SGWO	**1.88e-03**	**2.32e-03**	**1.32e-03**	**3.76e-03**

#### 6.4.2. SGWO for a large-scale optimization problem

To prove the scalability of SGWO in large-scale optimization problems [63], we conducted a comparative experiment under 1000 dimensions. The experimental information is the same as that in section 6.1.1. Table 14 shows the results of 8 algorithms in *f*_1_, *f*_2_, *f*_6_ and *f*_8_.

**Table 14:** Large-scale optimization results in 1000 dimensions.

*f*	Index	SCA	MFO	WOA	GWO	mGWO	CMAES	WSO	SGWO
*f*_1_	mean	2.92e+05	2.37e+06	5.70e-164	8.97e-10	2.73e-12	0.93675	4.08e+04	**0**
	max	4.64e+05	2.39e+06	1.71e-163	1.32e-09	3.52e-12	0.96727	4.36e+04	**0**
	min	7.53e+04	2.35e+06	1.19e-174	6.66e-10	1.46e-12	0.87738	3.61e+04	**0**
	std	1.98e+05	2.24e+04	0	3.73e-10	1.10e-12	0.05142	4.12e+03	**0**
*f*_2_	mean	Inf	Inf	6.74e-108	2.30e-05	3.98e-06	0.94723	5.18e+02	**0**
	max	Inf	Inf	1.99e-107	4.32e-05	6.66e-06	0.98153	5.23e+02	**0**
	min	Inf	Inf	1.25e-109	7.47e-06	3.53e-07	0.92390	5.15e+02	**0**
	std	NaN	NaN	1.14e-107	1.83e-05	3.25e-06	0.03034	4.11e+00	**0**
*f*_6_	mean	1.43e+03	1.43e+04	0	2.12e+01	1.10e-10	0.94289	5.79e+03	**0**
	max	2.34e+03	1.45e+04	0	2.67e+01	1.60e-10	0.96681	6.13e+03	**0**
	min	4.40e+02	1.39e+04	0	1.66e+01	7.45e-11	0.92584	5.57e+03	**0**
	std	9.55e+02	2.97e+02	0	5.04e+00	4.44e-11	0.02132	2.98e+02	**0**
*f*_8_	mean	4.08e+03	2.20e+04	0	3.02e-11	9.98e-03	0.92945	4.11e+02	**0**
	max	4.84e+03	2.24e+04	0	3.33e-11	2.99e-02	0.95888	4.44e+02	**0**
	min	3.16e+03	2.19e+04	0	2.79e-11	1.31e-13	0.90148	3.93e+02	**0**
	std	8.53e+02	2.77e+02	0	2.76e-12	1.72e-02	0.02873	2.88e+01	**0**

From Table 14, SGWO can still find theoretical optimal values in large-scale optimization problems. Compared to other algorithms, SCA and MFO failed on *f*_2_ and the results of WSO are also very poor on four functions. Therefore, SGWO is suitable for solving large-scale optimization problems and has strong stability.

### 6.5. SGWO-Elman comparative experiment

#### 6.5.1. Experimental information

(1) datasets information

To verify the performance of SGWO-Elman, we selected six benchmark datasets from the UCI (http://archive.ics.uci.edu/ml) database and did two groups of experiments. Because there are a few null values and characteristic indexes irrelevant to the study, the collected datasets were preprocessed. The processed data information was shown in Table 15. To eliminate the problem of dimensional inconsistency, normalization was carried out before the data was input into the prediction model. Table 16 shows the number of hidden layers for different datasets.

**Table 15:** Basic information about the six datasets.

No.	Name	Original sets	New sets	Output variables	Training set length	Testing set length	Reference
		Input variables					
D_1_	Air quality	15	14	1	6549	2808	[64]
D_2_	Wine quality white	12	11	1	3918	980	[65]
D_3_	QSAR aquatic toxicity	9	8	1	382	164	[66]
D_4_	QSAR fish toxicity	7	6	1	726	182	[67]
D_5_	Airfoil self-noise	5	5	1	1052	451	[68]
D_6_	Real estate valuation	7	5	1	290	124	[69]

**Table 16:** The number of hidden layers corresponding to different data sets.

Dataset	D_1_	D_2_	D_3_	D_4_	D_5_	D_6_
Hidden layers	15	6	10	12	10	10

(2) evaluation criteria

For performance testing, 10 runs have been performed in three comparative experiments. And experimental results are evaluated in terms of:

- Best of 10 MSEs of runs
- Worst of 10 MSEs of runs
- Mean of 10 MSEs of runs
- Standard deviation of 10 MSEs of runs

MSE is the minimum mean square error. MSE can evaluate the predictive performance of neural networks by comparing prediction errors. MSE metric was defined in Eq (20). The comparison methods of three experiments are as follows.

(3) comparison methods

For the first comparative experiment: we selected the SCA, MFO, sparrow search optimization algorithm (SSA) [35] and atom search optimization algorithm (ASO) [31] algorithms. They were fused into the Elman neural network to form SSA-Elman, MFO-Elman, ASO-Elman and SCA-Elman. These four optimization algorithms will be compared with SGWO-Elman. The parameters of all optimization algorithms were set to the same value.

For the second comparative experiment: we selected the traditional Elman neural network, standard back propagation neural network (BP), radial basis function neural network (RBF) [70], and generalized regression neural network (GRNN) [71]. The prediction effect of SGWO-Elman was determined by Elman. These four neural networks will be compared with SGWO-Elman.

For the third comparative experiment: we selected long short-term memory neural network (LSTM) [72] and RBF. They were fused into SGWO form SGWO-LSTM and SGWO-RBF. These two neural networks will be compared with SGWO-Elman. The parameters of all neural networks were set to the same value.

#### 6.5.2. Comparison experiments based on optimization strategy

Under the influence of SGWO performance, SGWO-Elman has better parameter optimization ability. To fairly analyze the optimization effect of SGWO on neural networks, Table 17 shows the comparison results of SGWO-Elman, SSA-Elman, MFO-Elman, ASO-Elman and SCA-Elman on six datasets. In Table 17, MSE metric can evaluate the predictive performance of neural networks by comparing prediction errors. MSE metric is the minimum mean square error, which was defined in Eq (20). Table 18 shows the prediction rankings of each algorithm on six datasets.

(1) prediction performance analysis

**Table 17:** Comparison of experimental results of the first group on MSE metric.

No.	Algorithm	Evaluating indicator			
		Mean	Std	Min	Max
D_1_	SSA-Elman	2.963500	6.244200	0.059000	19.76970
	MFO-Elman	39.34160	54.60130	0.081800	127.8225
	ASO-Elman	24.44260	31.86260	0.088300	73.22960
	SCA-Elman	0.088320	0.033895	0.039900	0.133800
	SGWO-Elman	**0.056700**	**0.017846**	**0.015800**	**0.073400**
D_2_	SSA-Elman	0.521780	0.018147	0.497400	0.553900
	MFO-Elman	0.514100	0.018646	0.493900	0.553800
	ASO-Elman	0.504390	0.019351	0.483800	0.543200
	SCA-Elman	0.505270	0.012983	0.484100	0.525600
	SGWO-Elman	**0.500940**	**0.010293**	**0.475300**	**0.509800**
D_3_	SSA-Elman	1.625600	0.070205	1.526600	1.699100
	MFO-Elman	1.586100	0.067165	1.511400	1.699300
	ASO-Elman	1.616300	0.086119	1.516600	1.792000
	SCA-Elman	1.535400	0.050547	1.454100	1.603300
	SGWO-Elman	**1.469900**	**0.029117**	**1.411700**	**1.514400**
D_4_	SSA-Elman	0.688310	0.017660	0.657660	0.716000
	MFO -Elman	0.700530	**0.012998**	0.679770	0.724840
	ASO-Elman	0.694010	0.019742	0.671500	0.731500
	SCA-Elman	0.681500	0.018423	0.657300	0.722300
	SGWO-Elman	**0.665920**	0.014590	**0.647380**	**0.692090**
D_5_	SSA-Elman	32.11200	3.293500	27.59640	36.62690
	MFO-Elman	31.03900	3.521600	27.75950	36.61040
	ASO-Elman	34.23630	6.551500	28.39370	45.85870
	SCA-Elman	28.88810	0.511490	28.04700	29.52820
	SGWO-Elman	**28.08570**	**0.477400**	**27.21390**	**29.13130**
D_6_	SSA-Elman	59.39180	2.240000	56.76840	63.48430
	MFO -Elman	57.10980	**1.895700**	54.10270	**60.24140**
	ASO-Elman	59.09030	2.573700	55.81940	64.62990
	SCA-Elman	57.93060	2.291600	55.56780	62.63620
	SGWO-Elman	**56.29050**	2.423300	**53.60270**	60.58750

**Table 18:** The prediction rankings of each algorithm on six datasets.

Algorithm	D_1_	D_2_	D_3_	D_4_	D_5_	D_6_	rate_sum	rank
SSA-Elman	3	5	5	3	4	5	25	5
MFO -Elman	5	4	3	5	3	2	22	3
ASO-Elman	4	2	4	4	5	4	23	4
SCA-Elman	2	3	2	2	2	3	14	2
SGWO-Elman	1	1	1	1	1	1	6	1

From Table 17, all results of SGWO-Elman are optimal except the std of D_4_ and D_6_, and significantly lower than other algorithms. This indicates that SGWO can reduce the Elman’s prediction error and improve the Elman prediction accuracy. Compared with other evolutionary strategies, SGWO algorithm based on an adaptive information interaction mechanism is an effective parameter optimization method. On D_1_, D_5_ and D_6_ datasets, SSA-Elman, MFO-Elman, ASO-Elman, and SCA-Elman have large errors. Through data analysis, it can be seen that D_1_ has a large amount of data and many data features, and the data features of D_5_ and D_6_ have weak correlations. Therefore, it is more complex to predict the three kinds of datasets. However, SGWO-Elman has a lower error on these three datasets, which indicates that SGWO-Elman is suitable for weakly correlated datasets and can show better prediction ability, stronger stability and higher robustness than other algorithms.

From Table 18, SGWO-Elman always ranks first on all datasets in prediction performance. SGWO-Elman > SCA-Elman > MFO-Elman > ASO-Elman > SSA-Elman. Therefore, for the prediction problem, SGWO has accurate prediction performance. And for the parameter optimization problem, SGWO has a better optimization effect.

**Fig 11:**
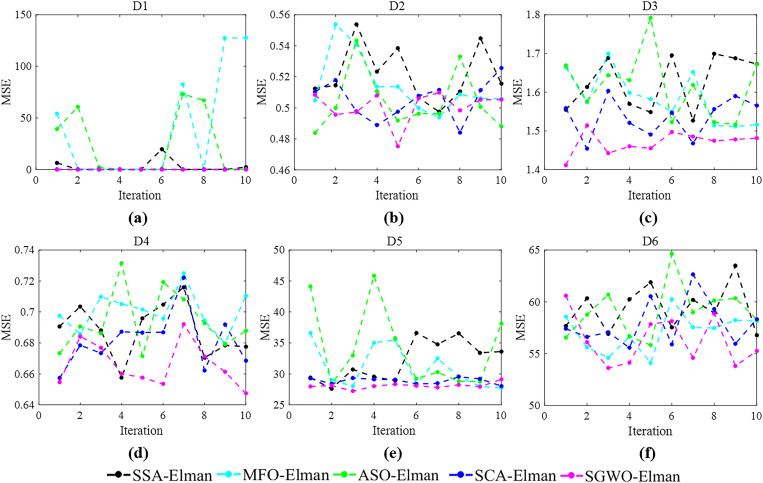
MSE of various datasets.

From Fig 11, the error of SGWO-Elman is lower than other algorithms on all datasets. On the D_1_, the errors of SGWO-Elman, SCA-Elman are close to zero, but ASO-Elman and MFO-Elman are very high, followed by SSA-Elman. On the D2—D_4_ datasets, the overall prediction error is low. Due to the limitations of D_5_ and D_6_, the MSE value of each algorithm is higher than other datasets. But the error distribution of SGWO-Elman is concentrated in D_5_. These show that SGWO-Elman has higher prediction performance and prediction accuracy and is suitable for most data. In practical engineering problems, using SGWO-Elman to predict can bring the greatest economic benefits to the project.

**Fig 12:**
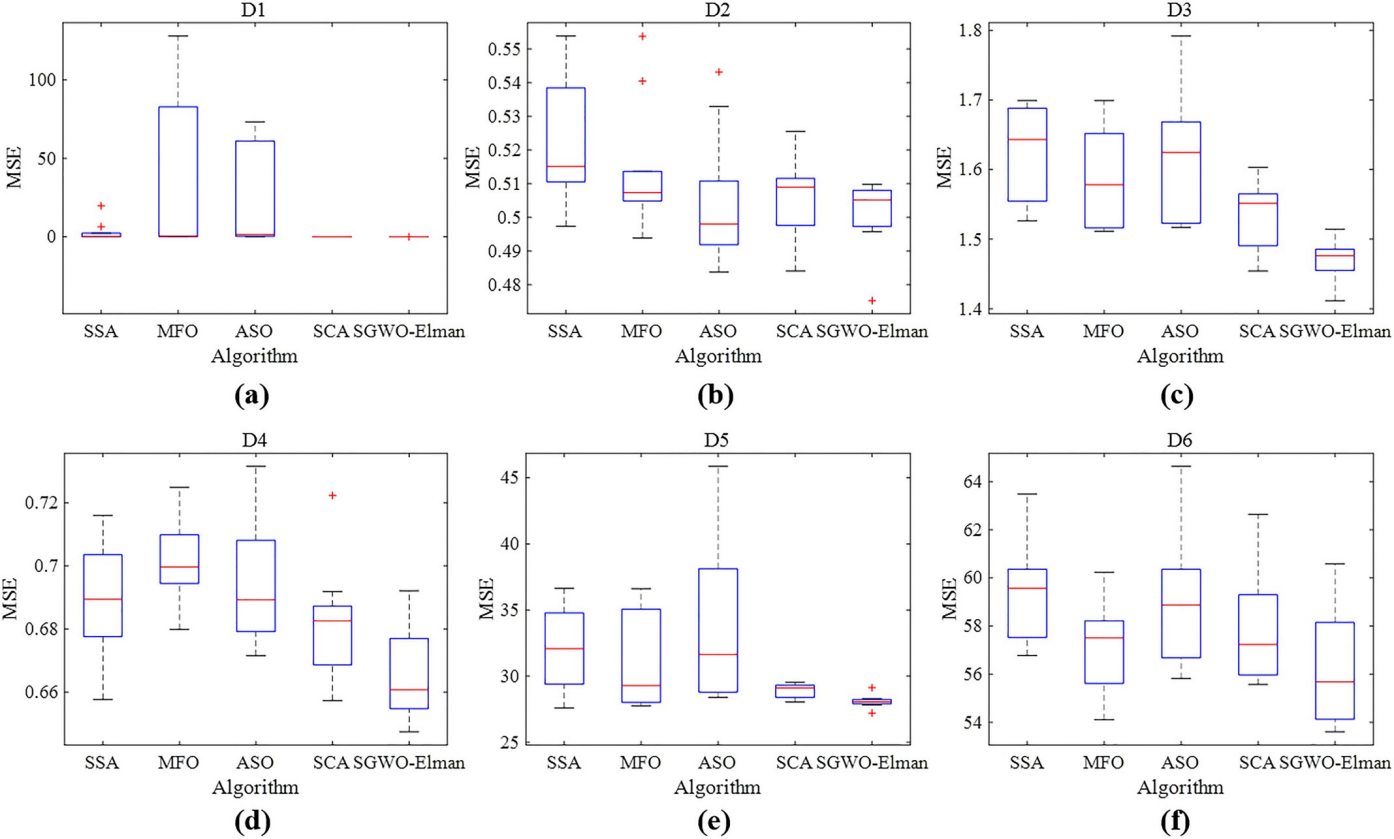
Boxplot of MSE in various datasets.

The result of the statistical analyses is presented on boxplots in Fig 12. From Fig 12, Compare with other algorithms SGWO-Elman has a lower median, and its lower quartile is close to the upper quartile in 6 kinds of datasets. There are almost no outliers in SGWO-Elman. Other algorithms have more outliers on D_1_, D_2_, D_4_ and D_5_. The results show that SGWO-Elman has higher prediction performance and stability than other algorithms. This fully verifies the good applicability of SGWO in Elman parameter optimization.

(3) training time analysis

**Fig 13:**
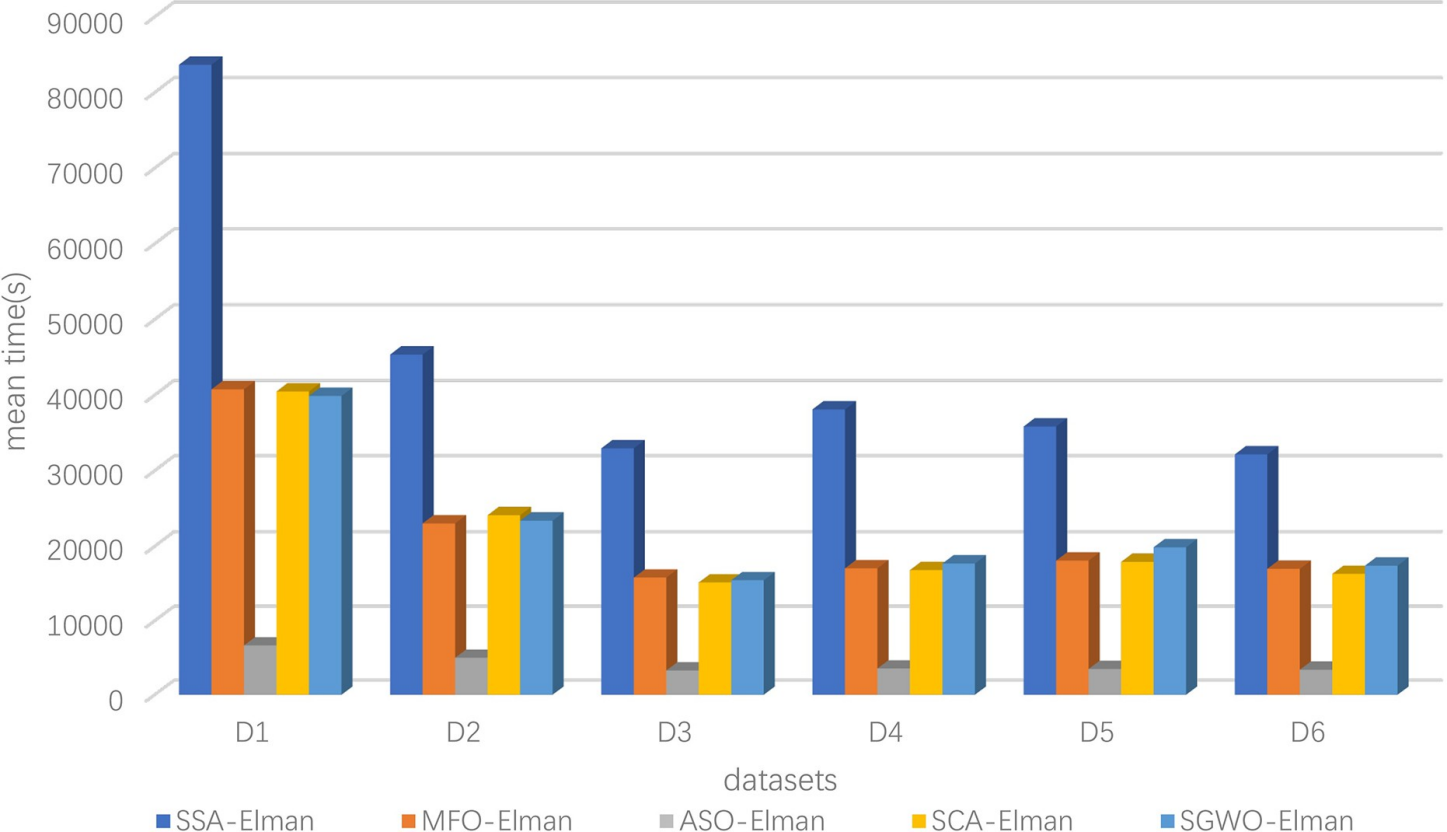
Mean training time of different algorithms.

To verify the running speed of SGWO-Elman, we tested five algorithms on six datasets. Table 19 records the average training time of each algorithm in 10 tests, and Fig 13 displays the histogram of Table 19.

**Table 19:** The mean training time(s) of each algorithm on six datasets.

Algorithm	D_1_	D_2_	D_3_	D_4_	D_5_	D_6_
SSA-Elman	83664	45348	32950	38093	35835	32148
MFO -Elman	40742	23015	15827	17076	18096	17008
ASO-Elman	**6661**	**4997**	**3279**	**3537**	**3481**	**3378**
SCA-Elman	40463	24099	15172	16840	17926	16329
SGWO-Elman	39876	23380	15578	17719	19859	17431

From Table 19 and Fig 13, it can be seen that ASO-Elman outperforms other algorithms in the average training on six datasets. SSA-Elman has the longest average training time. The average runtime of SGWO-Elman is not significantly different between SCA-Elman and MFO-Elman. Overall, the average training time of SGWO-Elman is at a medium level.

#### 6.5.3. Comparison experiments based on neural network

The prediction effect of SGWO-Elman was determined by Elman. To fairly analyze the prediction advantages of SGWO-Elman on various neural networks, it was compared with the traditional Elman neural network, standard BP neural network, the radial basis function neural network neural network (RBF) [70], and generalized regression neural network (GRNN) [71]. The experimental results are shown in Table 20.

**Table 20:** Comparison of experimental results of the second group.

No.	Algorithm	Evaluating indicator			
		Mean	Std	Min	Max
D_1_	BP	0.057070	0.0206790	**0.015200**	0.083400
	RBF	152.0866	14.967100	121.3891	167.2933
	GRNN	0.351810	0.1049400	0.204700	0.522800
	Elman	236.0867	223.98950	0.121400	665.4481
	SGWO-Elman	**0.056700**	**0.0178460**	0.015800	**0.073400**
D_2_	BP	0.525810	0.0090536	0.513600	0.545200
	RBF	0.546570	0.0105410	0.534300	0.564800
	GRNN	0.544650	**0.0068516**	0.534600	0.553400
	Elman	0.541710	0.0321310	0.490900	0.596200
	SGWO-Elman	**0.500940**	0.0102930	**0.475300**	**0.509800**
D_3_	BP	1.702800	0.2092400	1.467900	2.098000
	RBF	1.783200	0.0987620	1.638900	1.937500
	GRNN	1.957600	0.1797000	1.736000	2.298000
	Elman	1.522800	0.0717990	**1.408300**	1.622700
	SGWO-Elman	**1.469900**	**0.0291170**	1.411700	**1.514400**
D_4_	BP	0.762680	0.0727920	**0.584100**	0.849500
	RBF	0.772400	0.0523640	0.713200	0.827400
	GRNN	0.796070	0.0554430	0.701300	0.902900
	Elman	0.707660	0.0317400	0.646400	0.758600
	SGWO-Elman	**0.665920**	**0.0145900**	0.647380	**0.692090**
D_5_	BP	1147511	1954899.8	**0**	5354800
	RBF	301046	41305.584	2413500	358050
	GRNN	4075800	643986.16	302700	4783000
	Elman	4875110	1607992.9	1324300	6100300
	SGWO-Elman	**28.08570**	**0.4774000**	27.21390	**29.13130**
D_6_	BP	69.35730	14.546500	58.42460	107.9498
	RBF	58.42240	3.3258000	55.92840	67.18220
	GRNN	86.50280	5.5744000	75.29420	94.79750
	Elman	61.16360	**1.6401000**	58.96500	63.72170
	SGWO-Elman	**56.29050**	2.4233000	**53.60270**	**60.58750**

Comparing the prediction results of Elman with BP, RBF, and GRNN in Table 20. On the mean, Elman only has advantages in D_3_ and D_4_, it is inferior to BP and RBF in D_2_ and D_5_ respectively, and has a large error in D_1_ and D_6_. This indicates that the overall prediction performance of Elman needs to be improved. Std results demonstrate that Elman reaches the lowest error in the dataset more frequently than other algorithms, proving that Elman has a better stability. Elman and BP have the lowest error on three datasets in the min respectively. Elman has the lowest error in only three datasets in the max. Those indicate that Elman is prone to a large bias in predicting a certain sample point, and the prediction effect and robustness of Elman still need to be improved. According to the overall analysis, the comprehensive performance of Elman is slightly better than BP, RBF, and GRNN.

Comparing the prediction results of SGWO-Elman with other algorithms in Table 20. In terms of mean and max, SGWO-Elman maintains the lowest error in all datasets and ranks first. Std results show that SGWO-Elman has the lowest error in four datasets, indicating that SGWO significantly improves the prediction ability and robustness of Elman. In min, SGWO Elman performs best in D_2_ and D_6_, it ranks first in D_1_, second in D_3_ and D_5_, and third in D_4_, and values of SGWO Elman in D_5_ are far better than other algorithms by several orders of magnitude. Those indicate that SGWO-Elman can also produce better prediction ability for less relevant data sets.

Comprehensive analysis shows that SGWO-Elman has higher accuracy than other algorithms in general, which obviously improves the stability and predictive ability of Elman, making Elman demonstrate stronger memory function in neural networks. The neural evolution method based on SGWO is effective, and the neural network based on SGWO-Elman has higher prediction accuracy. SGWO-Elman plays a greater role in solving practical engineering problems with high complexity, ensuring the minimum misjudgment rate as far as possible to reduce the economic loss of engineering production.

**Fig 14:**
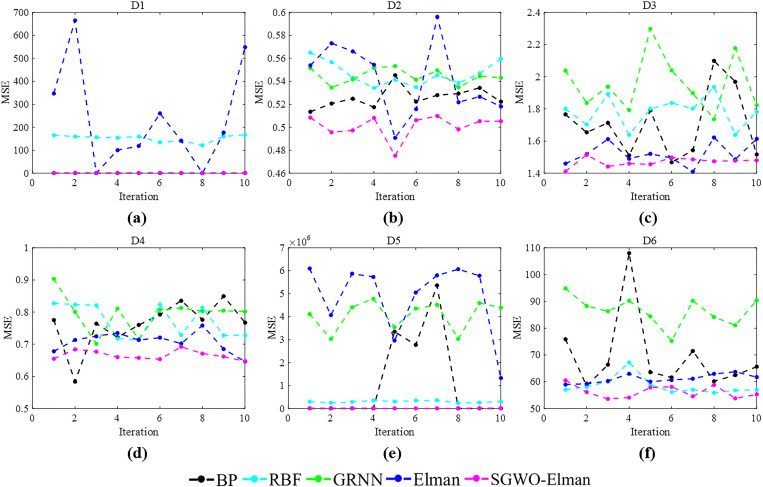
MSE of various datasets.

**Fig 15:**
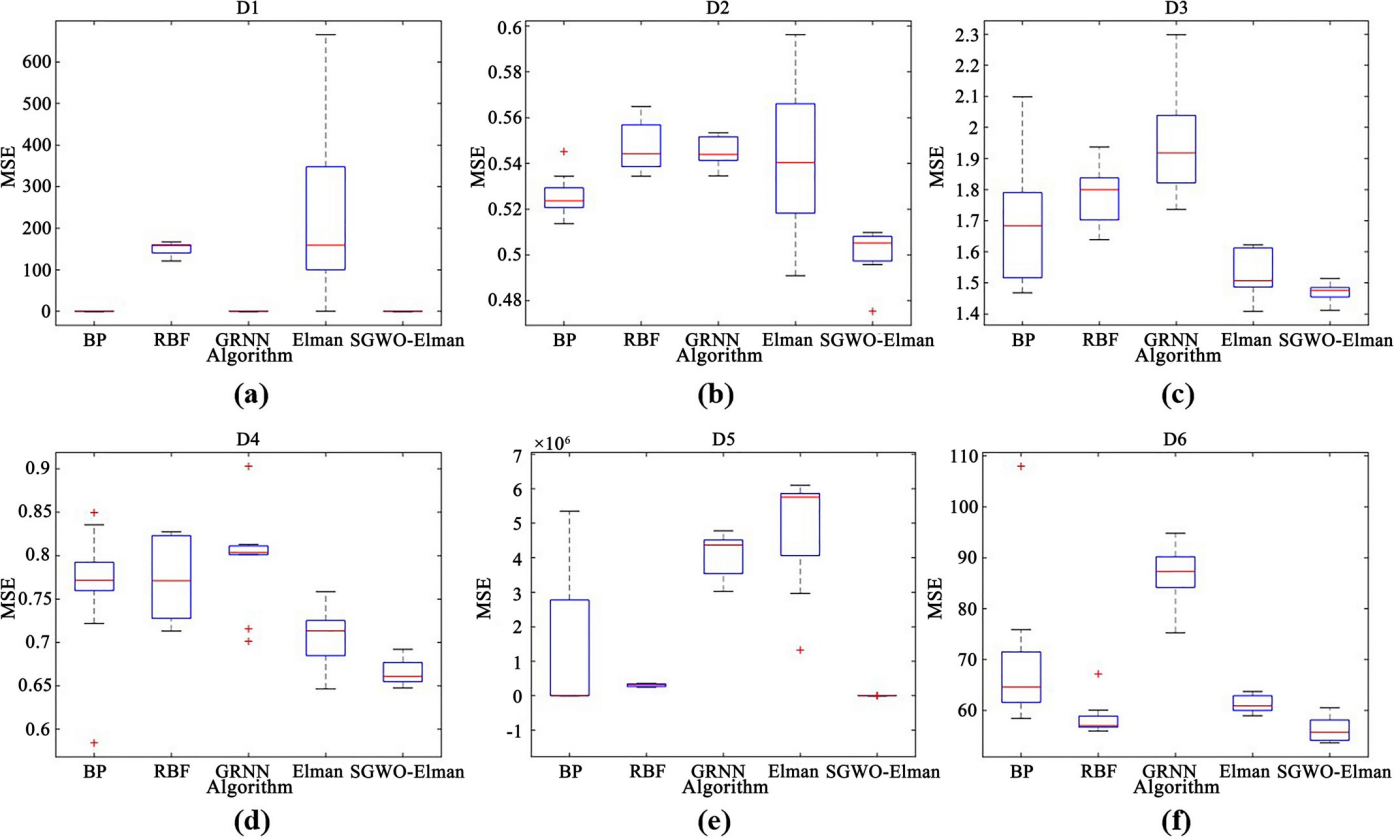
Boxplot of MSE in various datasets.

Figs 14 and 15 show MSE results and box diagrams respectively. From Fig 14, SGWO-Elman has a lower prediction error than other neural networks on all datasets. On D_1_, D_5_ and D_6_, although the error of other algorithms is very large, SGWO-Elman is still close to zero. This shows that neither evolutionary strategy nor the neural network applies to these datasets, but SGWO-Elman shows better prediction performance. On the D2—D_4_ datasets, SGWO-Elman still maintains better prediction accuracy. The overall analysis shows that the new neural network evolution strategy proposed in this paper can improve the shortcomings of traditional neural networks in parameter optimization. Elman based on SGWO is obviously superior to other neural networks and shows excellent prediction ability on most datasets. From Fig 15, the prediction errors of SGWO-Elman are lower than other neural networks, which indicates that the parameter optimization of the Elman neural network based on SGWO is effective. Compared with other algorithms, SGWO-Elman has no outliers on all datasets. In addition, SGWO-Elman centers the box graph on six datasets, which shows that parameters after multiple iterations can obtain a stable prediction effect relatively.

#### 6.5.4. SGWO for other neural networks

**Fig 16:**
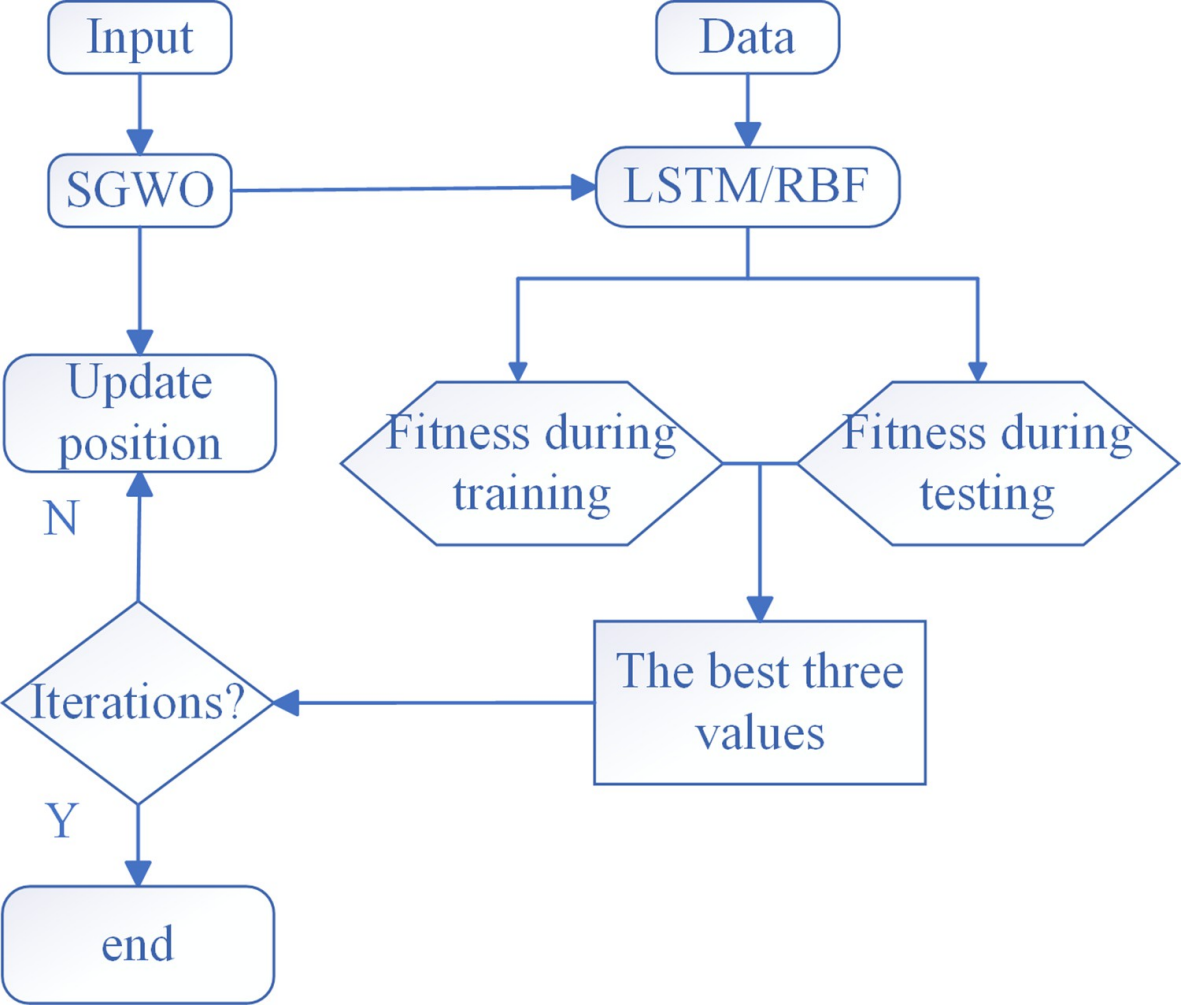
The implementation process of SGWO-RBF and SGWO-LSTM.

SGWO can extend to other types of neural networks, such as Long Short-Term Memory neural network (LSTM) and RBF. We incorporated SGWO into LSTM and RBF. The implementation steps for SGWO-LSTM and SGWO-RBF are shown in Fig 16. To verify the advantages of SGWO-Elman in prediction and optimization capabilities, we compared SGWO-Elman, SGWO-LSTM and SGWO-RBF.

Table 21 shows the experimental errors of the three algorithms on six datasets.

**Table 21:** The experimental errors of the three algorithms on six datasets.

No.	Algorithm	Evaluating indicator			
		Mean	Std	Min	Max
D_1_	SGWO-RBF	102.2839	1.9374610	20.30283	138.3902
	SGWO-LSTM	0.918010	**0.0109371**	0.372819	1.738190
	SGWO-Elman	**0.056700**	0.0178460	**0.015800**	**0.073400**
D_2_	SGWO-RBF	0.523921	0.0118980	0.509010	0.533840
	SGWO-LSTM	0.518103	**0.0023905**	0.492389	0.522803
	SGWO-Elman	**0.500940**	0.0102930	**0.475300**	**0.509800**
D_3_	SGWO-RBF	1.627490	0.0729174	1.429840	1.739483
	SGWO-LSTM	1.582891	0.0836184	1.429402	1.728204
	SGWO-Elman	**1.469900**	**0.0291170**	**1.411700**	**1.514400**
D_4_	SGWO-RBF	0.672784	0.0523640	**0.312931**	0.738914
	SGWO-LSTM	0.728921	0.0258159	0.528494	0.928471
	SGWO-Elman	**0.665920**	**0.0145900**	0.647380	**0.692090**
D_5_	SGWO-RBF	214566	3598.584	1456530	251045
	SGWO-LSTM	73.28191	3.429384	59.17382	109.2820
	SGWO-Elman	**28.08570**	**0.4774000**	**27.21390**	**29.13130**
D_6_	SGWO-RBF	**50.3890**	2.8291037	**45.39729**	57.93740
	SGWO-LSTM	53.2812	**0.9811907**	52.49104	**55.02971**
	SGWO-Elman	56.29050	2.4233000	53.60270	60.58750

From Table 21, it can be seen that the prediction error mean of SGWO-Elman on the six datasets is lower than SGWO-LSTM and SGWO-RBF. SGWO-LSTM has better prediction performance than SGWO-RBF. This not only indicates that SGWO as an optimization algorithm can significantly improve Elman’s prediction performance, but also SGWO-Elman’s prediction performance is higher than other neural networks. Meanwhile, SGWO-Elman has the lowest std value on the four datasets, which proves that SGWO-Elman has predictive stability.

## 7. Conclusion

In this study, the improved grey wolf optimizer was proposed and applied to the parameter optimization of the Elman neural network as an evolutionary strategy. Through theoretical analysis and numerical experiments, the optimization-seeking performance and prediction performance of the model was explored, and the following conclusions were obtained:

1. SGWO with an adaptive information interaction mechanism was proposed. This method used circle mapping to initialize the population, strengthened the information exchange among wolves in the channel through the Cauchy variant and the Golden-Sine algorithm, and updated the position of wolves with adaptive distance control weight.
2. Theoretical analysis proved that the global convergence probability of SGWO was 1, and that the experimental process of SGWO was a finite homogeneous Markov chain with absorbing states. Numerical experiments with 8 benchmark functions showed that SGWO can effectively improve convergence accuracy and optimization efficiency than other 6 algorithms.
3. The prediction performance of SGWO-Elman model was explored through comparative experiments. The results showed that SGWO-Elman model has good prediction accuracy, robustness and generalization performance. The index value in six datasets was better than the other evolutionary strategies and neural networks.

Although both the SGWO and SGWO-Elman proposed in this paper have better performance than the original algorithm, they still have some limitations. For example:

1. SGWO has no significant effect in solving practical optimization problems.
2. The training time of SGWO-Elman was higher than the other Elman based on optimization.
3. Due to the structural characteristics of the metaheuristic algorithm, the optimized neural network will have a dimensional disaster in complexity, which makes SGWO-Elman challenging in big data prediction and image recognition.

To address the above issues, we will conduct further research in the future, as follows.

1. In the future, we plan to improve the encircling mode of SGWO. We hope that this improved strategy is closer to the predatory behavior of wolves in nature. Meanwhile, we also plan to build a practical problem integrator, which will ensure that improved SGWO can be tested in integrator and improve the optimization ability in practical problems.
2. In the future, we plan to reduce the time complexity of SGWO-Elman. Since SGWO-Elman is the fusion of SGWO and Elman, and is influenced by SGWO algorithm, its time complexity is much higher than that of neural networks. Therefore, follow-up research will try to simplify the optimization processes in SGWO to reduce time and spatial complexity of the SGWO, thereby reducing the training and testing time of the SGWO-Elman.
3. In the future, we plan to build a preprocessing system based on SGWO-Elman. We hope that the system can extract early important features of big datasets and images. The system will reduce the complexity of the data entered into SGWO-Elman. Further, SGWO-Elman will be applied to predicate big data and recognize complex images by process system.

## Supporting information

S1 Data (ZIP)
