# Supplementary material for: Improved GWO and its application in parameter optimization of Elman neural network
Source: PLoS One. 2023 Jul 7;18(7):e0288071. doi: 10.1371/journal.pone.0288071 (PMC10328355; doi:10.1371/journal.pone.0288071)
Supplement: S1 Data — (ZIP) [file pone.0288071.s001.zip › SGWO-Elman and neural network.docx]

1000-D2

bp_Elman：0.52581

rbf-Elman：0.54657

grnn-Elman：0.54465

Elman：0.54171

SGWO-Elman：0.50094

bp_Elman：0.0090536

rbf-Elman：0.010541

rgnn-Elman：0.0068516

Elman：0.032131

SGWO-Elman：0.010293

bp_Elman：0.5136

rbf-Elman：0.5343

rgnn-Elman：0.5346

Elman：0.4909

SGWO-Elman：0.4753

bp_Elman：0.5452

rbf-Elman：0.5648

rgnn-Elman：0.5534

Elman：0.5962

SGWO-Elman：0.5098

1000-D3

bp_Elman：1.7028

rbf-Elman：1.7832

grnn-Elman：1.9576

Elman：1.5228

SGWO-Elman：1.4699

bp_Elman：0.20924

rbf-Elman：0.098762

rgnn-Elman：0.1797

Elman：0.071799

SGWO-Elman：0.029117

bp_Elman：1.4679

rbf-Elman：1.6389

rgnn-Elman：1.736

Elman：1.4083

SGWO-Elman：1.4117

bp_Elman：2.098

rbf-Elman：1.9375

rgnn-Elman：2.298

Elman：1.6227

SGWO-Elman：1.5144

1000-D4

bp_Elman：0.76268

rbf-Elman：0.7724

grnn-Elman：0.79607

Elman：0.70766

SGWO-Elman：0.66592

bp_Elman：0.072792

rbf-Elman：0.052364

rgnn-Elman：0.055443

Elman：0.03174

SGWO-Elman：0.01459

bp_Elman：0.5841

rbf-Elman：0.7132

rgnn-Elman：0.7013

Elman：0.6464

SGWO-Elman：0.64738

bp_Elman：0.8495

rbf-Elman：0.8274

rgnn-Elman：0.9029

Elman：0.7586

SGWO-Elman：0.69209

1000D5

bp_Elman：1147511

rbf-Elman：301046

grnn-Elman：4075800

Elman：4875110

SGWO-Elman：28.0857

bp_Elman：1954899.8208

rbf-Elman：41305.584

rgnn-Elman：643986.1627

Elman：1607992.9025

SGWO-Elman：0.4774

bp_Elman：0

rbf-Elman：241350

rgnn-Elman：3027000

Elman：1324300

SGWO-Elman：27.2139

bp_Elman：5354800

rbf-Elman：358050

rgnn-Elman：4783000

Elman：6100300

SGWO-Elman：29.1313

1000D6

bp_Elman：69.3573

rbf-Elman：58.4224

grnn-Elman：86.5028

Elman：61.1636

SGWO-Elman：56.2905

bp_Elman：14.5465

rbf-Elman：3.3258

rgnn-Elman：5.5744

Elman：1.6401

SGWO-Elman：2.4233

bp_Elman：58.4246

rbf-Elman：55.9284

rgnn-Elman：75.2942

Elman：58.965

SGWO-Elman：53.6027

bp_Elman：107.9498

rbf-Elman：67.1822

rgnn-Elman：94.7975

Elman：63.7217

SGWO-Elman：60.5875
