# Supplementary material for: Improved GWO and its application in parameter optimization of Elman neural network
Source: PLoS One. 2023 Jul 7;18(7):e0288071. doi: 10.1371/journal.pone.0288071 (PMC10328355; doi:10.1371/journal.pone.0288071)
Supplement: S1 Data — (ZIP) [file pone.0288071.s001.zip › SGWO-Elman and other optimization algorithms.docx]

1

ssa_Elman：2.9635

mfo-Elman：39.3416

aso-Elman：24.4426

sca_Elman：0.08832

SGWO-Elman：0.0567

ssa_Elman：6.2442

mfo-Elman：54.6013

aso-Elman：31.8626

scaElman：0.033895

SGWO-Elman：0.017846

ssa_Elman：0.059

mfo-Elman：0.0818

aso-Elman：0.0883

scaElman：0.0399

SGWO-Elman：0.0158

ssa_Elman：19.7697

mfo-Elman：127.8225

aso-Elman：73.2296

scaElman：0.1338

SGWO-Elman：0.0734

2

ssa_Elman：0.52178

mfo-Elman：0.5141

aso-Elman：0.50439

sca_Elman：0.50527

SGWO-Elman：0.50094

ssa_Elman：0.018147

mfo-Elman：0.018646

aso-Elman：0.019351

scaElman：0.012983

SGWO-Elman：0.010293

ssa_Elman：0.4974

mfo-Elman：0.4939

aso-Elman：0.4838

scaElman：0.4841

SGWO-Elman：0.4753

ssa_Elman：0.5539

mfo-Elman：0.5538

aso-Elman：0.5432

scaElman：0.5256

SGWO-Elman：0.5098

3

ssa_Elman：1.6256

mfo-Elman：1.5861

aso-Elman：1.6163

sca_Elman：1.5354

SGWO-Elman：1.4699

ssa_Elman：0.070205

mfo-Elman：0.067165

aso-Elman：0.086119

scaElman：0.050547

SGWO-Elman：0.029117

ssa_Elman：1.5266

mfo-Elman：1.5114

aso-Elman：1.5166

scaElman：1.4541

SGWO-Elman：1.4117

ssa_Elman：1.6991

mfo-Elman：1.6993

aso-Elman：1.792

scaElman：1.6033

SGWO-Elman：1.5144

4

ssa_Elman：0.68831

mfo-Elman：0.70053

aso-Elman：0.69401

sca_Elman：0.6815

SGWO-Elman：0.66592

ssa_Elman：0.01766

mfo-Elman：0.012998

aso-Elman：0.019742

scaElman：0.018423

SGWO-Elman：0.01459

ssa_Elman：0.65766

mfo-Elman：0.67977

aso-Elman：0.6715

scaElman：0.6573

SGWO-Elman：0.64738

ssa_Elman：0.716

mfo-Elman：0.72484

aso-Elman：0.7315

scaElman：0.7223

SGWO-Elman：0.69209

5

ssa_Elman：32.112

mfo-Elman：31.039

aso-Elman：34.2363

sca_Elman：28.8881

SGWO-Elman：28.0857

ssa_Elman：3.2935

mfo-Elman：3.5216

aso-Elman：6.5515

scaElman：0.51149

SGWO-Elman：0.4774

ssa_Elman：27.5964

mfo-Elman：27.7595

aso-Elman：28.3937

scaElman：28.047

SGWO-Elman：27.2139

ssa_Elman：36.6269

mfo-Elman：36.6104

aso-Elman：45.8587

scaElman：29.5282

SGWO-Elman：29.1313

6

ssa_Elman：59.3918

mfo-Elman：57.1098

aso-Elman：59.0903

sca_Elman：57.9306

SGWO-Elman：56.2905

ssa_Elman：2.24

mfo-Elman：1.8957

aso-Elman：2.5737

scaElman：2.2916

SGWO-Elman：2.4233

ssa_Elman：56.7684

mfo-Elman：54.1027

aso-Elman：55.8194

scaElman：55.5678

SGWO-Elman：53.6027

ssa_Elman：63.4843

mfo-Elman：60.2414

aso-Elman：64.6299

scaElman：62.6362

SGWO-Elman：60.5875
